# Assessing Nonbonded Aggregates Populations: Application to the Concentration-Dependent IR O–H Band of Phenol

**DOI:** 10.1021/acs.jctc.5c00281

**Published:** 2025-04-14

**Authors:** J. Pablo Gálvez, José Zúñiga, Javier Cerezo

**Affiliations:** † Departamento de Química Física, 16751 Universidad de Murcia, 30100 Murcia, Spain; ‡ Departamento de Química and Institute for Advanced Research in Chemical Sciences (IAdChem), Universidad Autónoma de Madrid, 28049 Madrid, Spain

## Abstract

In this work, we present two alternative computational strategies to determine the populations of nonbonded aggregates. One approach extracts these populations from molecular dynamics (MD) simulations, while the other employs quantum mechanical partition functions for the most relevant minima of the multimolecular potential energy surfaces (PESs), identified by automated conformational sampling. In both cases, we adopt a common graph-theory-based framework, introduced in this work, for identifying aggregate conformations, which enables a consistent comparative assessment of both methodologies and provides insight into the underlying approximations. We apply both strategies to investigate phenol aggregates, up to the tetramer, at different concentrations in phenol/carbon tetrachloride mixtures. Subsequently, we simulate the concentration-dependent OH stretching IR region by averaging the harmonic Infrared (IR) spectra of aggregates using the populations predicted by each strategy. Our results indicate that the populations extracted from MD trajectories yield OH stretching signals that closely follow the experimental trends, outperforming the spectra from populations obtained by systematic conformational searches. Such a better performance of MD is attributed to a better description of the entropic contributions. Moreover, the proposed protocol not only successfully addresses a very challenging problem but also offers a benchmark to assess the accuracy of the intermolecular force fields.

## Graphical abstract

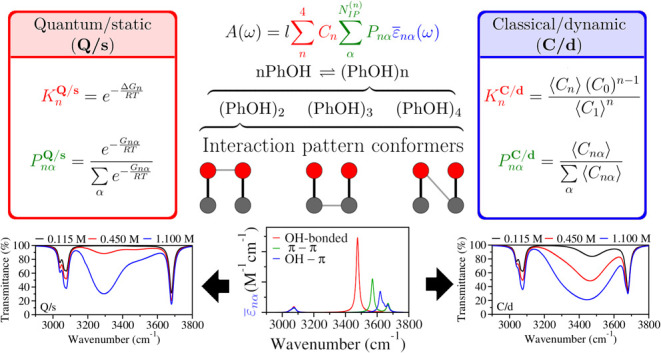

## 1 Introduction

Noncovalent interactions, such as dispersion, hydrogen or halogen bonding, and π–π stacking, play a pivotal role in the condensed phase. These interactions are fundamental to the supramolecular organization of biologically and technologically relevant systems, including protein structures, DNA base-pairing, and liquid crystals.^1^ Detailed understanding of these interactions is, therefore, crucial for advancements in material design, drug discovery, or protein folding.

Molecular aggregates, which are held together by such nonbonded interactions, may present a remarkable challenge, as their relative populations are governed by a delicate balance of enthalpic and entropic contributions. Indeed, predicting these populations requires (i) an accurate description of nonbonded interactions, which determine the enthalpic component, and (ii) a thorough accounting of the extensive range of conformers arising from complex potential energy surfaces (PESs) with numerous energetically accessible minima, which govern the entropic contribution.

Accurate modeling of nonbonded interactions necessitates electronic structure methods that incorporate nonlocal correlation effects, often requiring computationally demanding post-Hartree–Fock techniques. However, density functional theory (DFT), enhanced by suitable correction schemes such as Grimme’s empirical dispersion corrections,^2,3^ provides a computationally feasible alternative for systems dominated by noncovalent interactions. Combined with implicit solvent models,^4^ DFT has significantly expanded its applicability to biologically and technologically important systems. Still, such approaches become impractical for large systems requiring numerous potential evaluations, such as in molecular dynamics (MD) simulations. In such cases, interatomic interactions are typically described using empirical molecular mechanics (MM) potentials, where nonbonded interactions are represented by Coulombic terms for electrostatics and Lennard–Jones (LJ) potentials for van der Waals forces. While polarizable force fields offer improved descriptions,^5,6^ simpler point charges and LJ potentials still remain the standard in MD simulations, whose quality can be improved by reparameterizing them against quantum mechanical (QM) energy databases.^7^

Moving to the exploration of conformational flexibility, automated conformational searches are commonly employed to identify the most relevant solute configurations,^8,9^ particularly when interactions are described at QM level. The PES around these minima is typically approximated using harmonic models, with specific adaptations available to account for nonharmonic regions, such as torsional degrees of freedom. Solvent effects are generally included through implicit solvation models, and aggregate populations are ultimately derived by constructing partition functions that incorporate quantum mechanical treatment of vibrations.

Alternatively, classical MD simulations, which explicitly represent the solvent, provide a highly effective approach for exploring the configurational space of systems, encompassing both the solute and solvent. However, few studies have applied MD simulations to directly calculate the populations of clusters of varying sizes within the context of organic aggregates.^10–13^ Unbiased MD simulations have primarily been limited to obtaining statistical insights into aggregate sizes and structural characteristics. These analyses typically involve tracking the average number of noncovalent interactions throughout the trajectory,^11,14–19^ computing radial or pair distribution functions,^20–23^ or monitoring the evolution of structural parameters.^24,25^

A critical step in these strategies is the identification of unique conformers from the vast number of molecular configurations typically generated. This challenge can be effectively addressed using graph theory tools. Specifically, adjacency matrix analysis has been successfully applied to classify molecular configurations into different topological conformers in MD snapshots of individual solute molecules in solution^26,27^ or to distinguish topologically distinct, QM-optimized isomers.^28^ However, a comprehensive top-down approach that identifies individual aggregates coexisting in solution potentially within the same snapshot, in terms of their isomorphic conformations, is still lacking. Such a framework is essential for accurately calculating populations in systems involving thousands of particles.

A notable strength of MD approaches for thermodynamic predictions is their independence from prior knowledge of aggregate structures, unlike strategies that compute partition functions that require the identification of the relevant minima. Nevertheless, several challenges persist for MD-based approaches. Accurate determination of population distributions requires a dynamic evaluation of chemical identities at each simulation step, which typically relies on geometric criteria that may involve some arbitrariness. Furthermore, for species present at low concentrations, achieving sufficient conformational sampling demands extended simulation times and large system sizes. In contrast, first-principles quantum chemistry calculations are well suited to these scenarios, employing efficient reaction equilibrium algorithms to compute species concentrations without the need for extensive sampling.^29–31^ Still, a comprehensive and reliable framework to compare the performance of MD-based and partition-function-based approaches remains an open challenge.

The effects of noncovalent interactions, which influence the populations of aggregates, are ultimately manifested in the spectroscopic signals of the system. Infrared (IR) spectra, in particular, contain exquisite information about microscopic characteristics and provide detailed insights into key noncovalent interactions, notably hydrogen bonds.^32,33^ Recent advances have reported simulations of IR and vibrational circular dichroism (VCD) spectra based on aggregate populations for pure solvents^34–42^ and solvent mixtures with various solutes.^43–52^ These approaches involve averaging the spectra of optimized clusters and calculating their populations using either the quantum cluster equilibrium theory^53–57^ or the clusters-in-a-liquid framework.^43^ While most studies focus on the fingerprint region, simulations of the O–H stretching region for hydrogen-bonded aggregates^36,37,39,40,42,52^ have revealed limitations in addressing the challenging broadband that is observed. The formation of hydrogen bonds significantly affects the vibrational spectrum, shifting the O–H stretching bands of the acceptor to lower wavenumbers. Indeed, this band is highly sensitive to the number and arrangement of hydrogen bonds, whether intra- or intermolecular. Consequently, these characteristics make the simulations of such an O–H stretching region (3100–3800 cm^–1^) a stringent test to compare populations derived from MD and conformational search-based methods.

Building on these ideas, we first develop two methodologies to evaluate the populations of nonbonded aggregates—one based on the analysis of MD trajectories and the other on conformational searches followed by a statistical thermodynamic treatment using model potential energy surfaces, and subsequently assess their accuracy by simulating the IR spectrum, focusing on the O–H stretching region. Our methodologies are applied to phenol/carbon tetrachloride mixtures, which presents marked changes in the O–H IR band with concentration. In these mixtures, phenol monomers coexist with various aggregates, whose distinct peaks in the 3100–3800 cm^–1^ range reflect the structure and conformational landscape of the system.^58^ Self-association of phenol in CCl_4_ indeed remains a challenging problem and, despite extensive experimental studies over the past decades,^59–71^ no general consensus exists regarding the degree of aggregation or the structures of the resulting clusters.

In this study, we analyze the populations of phenol clusters in solution through atomistic simulations, extending our analysis to tetramers. The two developed strategies are applied to compute the populations of phenol aggregates, which are eventually used to simulate the average IR spectra at varying phenol/CCl_4_ ratios, focusing on the challenging O–H broadband. Our results offer valuable insights into the strengths and limitations of both approaches in capturing enthalpic and entropic contributions to aggregate formation.

This paper is organized as follows: Section 2 provides a detailed description of the newly developed approaches for determining aggregate populations and outlines the method for calculating averaged IR spectra. Section 3 presents the computational details. Section 4 discusses the main findings, and the conclusions are presented in Section 5.

## 2 Theoretical Methods

This section begins by introducing the graph-theory-based approach used to identify equivalent conformers, which applies to both methodologies for determining aggregate populations. The two strategies, termed **Q/s** and **C/d**, are then described in detail, eventually providing a concise comparison between them. Finally, we outline the procedure to compute IR spectra based on the populations derived from these two approaches.

### 2.1 Unique Classification of Conformers Based on Graph Theory

In this study, we have developed and implemented a systematic procedure for identifying and classifying macromolecular aggregates based on powerful algorithms of graph theory. Our method is primarily intended to analyze MD trajectories, which usually contain thousands of particles. Indeed, their analysis is far from straightforward, which explains why traditional MD analysis tools do not directly provide information about these aggregates that may appear for short periods of time during the simulation. Moreover, as we will show, the top-down nature of our approach makes it generally applicable to any set of configurations, for instance, those arising from a systematic conformational search.

A molecular graph is a two-dimensional (2D) representation of a molecule where atoms and bonds are typically simplified as vertices and edges of the graph, respectively. Graphs can be represented in matrix form, leading to computationally efficient methods to extract information from the system. In this work, we resort to the adjacency matrix, which contains topological information, i.e., the covalent and noncovalent bonds within the atoms. The knowledge of this matrix at each snapshot ultimately provides us with the information about the isolated molecular clusters present during the simulation.

In the initial step, the analysis of the adjacency matrix of the entire system determines the number of aggregates of each size, *n*. This is achieved by transforming the adjacency matrix of the system into a block-diagonal form, where each diagonal block represents the individual adjacency matrix of a distinct aggregate in the system. The dimensions of this sub-matrix determine the size of the aggregate. In this way, from the number of blocks of identical dimensions and the total number of blocks, we can calculate the population of aggregates of varying sizes.

For the sake of simplicity, we consider only the connectivity between the functional groups of the molecules, represented as the nodes in an undirected graph. In the case of phenol, we focus on the hydroxyl group (OH) and the phenyl group (π) of the monomer. Aggregate configurations, up to tetramers, are classified according to their interaction patterns, i.e., the topology according to the functional groups. Although interactions are determined based on the position (or center of mass) of atoms (or groups of atoms), we employ a simplified representation based on functional groups to classify the conformations explored during the simulation according to their interaction patterns in topologically distinct isomers. While using this simplified graphical representation does not retain information about the specific donor and acceptor molecules forming, e.g. a hydrogen bond, it offers a streamlined approach for evaluating the effectiveness and limitations of this clustering-like method in analyzing molecular configurations.

The interaction patterns considered for the phenol dimer and the associated molecular graphs are illustrated in Figure 1. Hereafter, we refer to the set of geometric configurations that can be associated with the same interaction pattern as conformers of a given isomer. One interaction (edge) between hydroxyl groups corresponds to a hydrogen bond (H-bond) between them. The presence of an edge between two phenyl groups can indicate either a pure π···π interaction between the aromatic rings or C–H···π interactions between a C–H bond in one molecule and the π-electron cloud of an aromatic ring. Finally, OH–π bonded isomers correspond to either an H-bond between an aromatic hydrogen atom and the hydroxyl group or a hydroxyl proton with the π-electron cloud.

**Figure 1:**
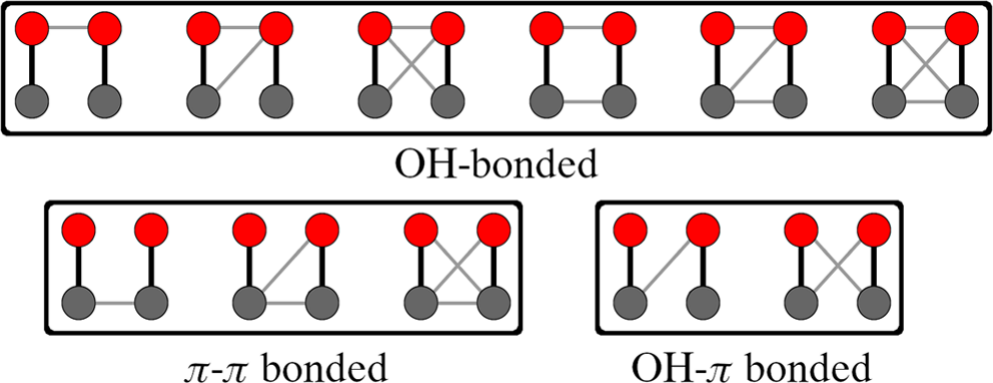
Molecular graphs used to classify interaction patterns in phenol dimers. Red and gray circles represent hydroxyl and phenyl groups, respectively. Black edges indicate the existence of covalent bonds between atoms within each node, while gray edges represent noncovalent interactions.

Naturally, the number of possible molecular graphs increases substantially in higher-order clusters. To simplify our analysis, trimer and tetramer configurations are classified to attend to the topology of the H-bonds among molecules. If no H-bond is present, they are categorized as either π–π or OH–π bonded clusters, depending on whether any monomers interact solely through OH···π interactions or not. This results in five distinct interaction patterns for trimers and ten for tetramers, which are schematized in Figures S15 and S16 in the Supporting Information (SI) and listed in the first column of Table 1.

In our approach, intermolecular interactions are defined based on specific geometrical criteria (detailed information on the selection of the criteria used to determine the noncovalent interactions in the system is provided in Section A of the SI). Two phenol molecules are considered to form a hydrogen bond when the O···H distance is less than 2.6 Å and the O···H–O angle is between 135 and 180°. The π-electronic clouds of two phenyl groups are considered to interact when the distance between the centers of mass of the carbon atoms in each molecule is less than 4.0 Å. C–H···O interactions are present if the H···O distance is lower than 2.8 Å, and the angle with the adjacent C atom is between 120 and 180°. Finally, X–H···π interactions are defined when the H···π distance is lower than 3.0 Å, with a C–H···π or O–H···π angle in the range of 120–180° or 135°–180°, respectively. The effect on the populations of using different thresholds has been proven to be rather limited, as shown in the SI (Section D), which supports the robustness of our approach.

This protocol can be applied to the snapshots generated through MD simulations, which may include all or just part of the interaction patterns. The total population would be given by the average over all snapshots, which would generally include all of the interaction patterns for sufficiently long trajectories. The same protocol can be applied to an ensemble of configurations generated through a systematic conformational search. In this case, the number of interaction patterns identified will depend on the extent of the automated search. All algorithms described here have been implemented in aggregate,^72^ a code developed by one of us to identify and classify interaction patterns.

### 2.2 Quantum/Static (**Q/s**) Approach

The **Q/s** strategy starts from a systematic conformational search to locate all possible conformers for monomers to tetramers, which are subsequently analyzed by means of statical thermodynamics, in line with quantum cluster equilibrium theory^53–57^ or the clusters-in-a-liquid framework.^43^ Here, we used *AutoMeKin* ^73–75^ software, with some adaptations as explained in Section 3.2, to generate the conformational space. This results in a set of unique isomers for each aggregation size, containing 1 monomer, 38 dimers, 174 trimers, and 144 tetramers. Each of them is optimized, and their second derivatives (frequencies) are subsequently computed at the DFT/PCM level (see Section 3.1 for details).

All of the aforementioned isomers may be grouped into macroscopically differentiable conformers. Specifically, we here adopt the interaction-pattern conformers identified with the graph-theory tool described in Section 2.1, aggregate, in terms of hydrogen bonds, π–π interactions, and π–OH contacts. Therefore, each interaction-pattern conformer includes, in general, several isomers. We then account for such a multimolecular description of the conformer treating the molecular system as an equilibrated mixture of all of the minimum-energy structures with a total free energy given by eq 1 ^31,76–78^

1 $$G\left(\left\{X\right\}\right)=G\left(X_{ref}\right)-\mathrm{RT}\ln\left[\sum_{X_{i}\in \left\{X\right\}}m_{i}e^{-\left(\Delta G\left(X_{i}\right)/\mathrm{RT}\right)}\right]$$

where the sum runs over the set of all energy-minimized molecular conformations (isomers) that are characterized by a given interaction pattern, {**X**}. In the previous expression, Δ*G*(*X*_*i*_) = *G*(*X*_*i*_) – *G*(*X*_ref_), with *X*_ref_ being some arbitrarily chosen reference configuration and *G*(*X*_*i*_) the absolute free energy corresponding to the *i*th conformation. *m*_*i*_ is the number of equivalent energy wells in the PES of the system compatible with species *i* (conformers and rotamers), which can be obtained by using either a direct counting method or automatic conformational search algorithms. When dealing with noncovalent complexes, this PES degeneracy must also account for the indistinguishability of monomer molecules.^78^

At this point, we still have to determine the Gibbs free energy of each individual isomer (*G*(*X*_*i*_)). This corresponds to the standard free energy at temperature *T* and pressure *p, G­(X_i_) ≡ G*_i_^*^(*T*, *p*), of solute *i*, which is determined as the sum of the following terms^79^

2 $$G_{i}^{*}\left(T,p\right)=E_{i,\mathrm{gas}}^{\mathrm{elec}}+\Delta G_{i,\mathrm{solv}}^{*}\left(T,p^{{}^{\circ}}\right)+\delta G_{i,\mathrm{VRT}}^{*}+\mathrm{RT}\ln\left(\frac{M_{\mathrm{solv}}C^{{}^{\circ}}}{1000\rho_{\mathrm{solv}}\left(T,p\right)}\right)$$

In this equation, *E*_*i*,gas_^elec^ is the gas-phase total electronic energy of the solute and Δ*G*_*i*_^solv^(*T*, *p*°) is its solvation Gibbs free energy (defined to transfer a solute from an ideal gas at a concentration of 1 mol L^–1^ to an ideal solution at a concentration of 1 mol L^–1^). The third term of this equation is usually termed the vibrational–rotational–translational contribution to the gas-phase Gibbs free energy. It can be written as $\delta G_{i,\mathrm{VRT}}^{*}=\delta G_{i,\mathrm{VRT}}^{{}^{\circ}}+\mathrm{RT}\ln\left(\frac{\mathrm{RT}}{p^{{}^{\circ}}}\right)$ , and it includes the free energy change associated with bringing the solute from its ideal gas reference state at *p*° = 1 bar to a density of unit molarity,^80^ i.e., a reference state concentration of 1 mol L^–1^. Note that omitting this factor would lead to a systematic error of 1.89 kcal mol^–1^ at 298.15 K to the absolute free energies.^81^ The last term involves the density and, despite being systematically omitted in the literature, has a notable impact on the free energies at high temperatures, especially for nonaqueous solvents, as shown by Noroozi and Smith.^80^

Eq 2 provides the absolute free energies in solution at the standard state of 1 mol L^–1^ used in this work. Thermal contributions to solution-phase free energies (δG*VRT) are computed by referencing the partition function to the ground-state electronic energy, which inherently includes the zero-point vibrational energy (ZPE). These contributions are calculated using ideal gas partition functions under the quasi-rigid rotor harmonic oscillator (quasi-RRHO) approximation,^82,83^ employing scaled^84^ solution-phase harmonic vibrational frequencies, with the temperature and pressure fixed at 298.15 K and 1 bar, respectively. The vibrational frequencies were scaled for thermal correction calculations using scaling factors derived in this work from experimentally measured fundamental frequencies of the phenol monomer in the gas phase and in CCl_4_ solution (see Section C in the SI).

Once the free energies of each interaction pattern, α, are evaluated, we can now obtain the populations, which we here define as the molar fraction over the total amount of the aggregate with the same size, *n*, as

3 $$P_{n\alpha }^{Q/s}=\frac{e^{-\left(G_{n\alpha }/\mathrm{RT}\right)}}{\sum_{\alpha }e^{-\left(G_{n\alpha }/\mathrm{RT}\right)}}$$

where *G*_*n*α_ is computed according to eq 1 including all of the isomers classified within the interaction pattern α.

Eqation [Disp-formula eq3] provides the populations of each conformer relative to the aggregate of size *n*. The complete elucidation of the system composition thus requires the macroscopic concentration of each aggregate, which is computed from the self-association equilibria

4 $$n\mathrm{PhOH}⇌{\left(\mathrm{PhOH}\right)}_{n}$$

The thermodynamic constant of the above equilibrium is calculated from the global free energy of the aggregate of size *n*

5 $$K_{n}^{Q/s}=e^{-\left(\Delta G_{n}/\mathrm{RT}\right)}$$

where the free energies include the contribution of all interaction-pattern conformers of this size through eq 1.

As dictated by the self-aggregation equilibrium, eq 4, the concentrations of the monomer (*C*_1_) and that of the *n*-th aggregate (*C*_*n*_) at equilibrium must obey

6 $$K_{n}=\frac{C_{n}{\left(C^{{}^{\circ}}\right)}^{n-1}}{{\left(C_{1}\right)}^{n}}$$

where we have included the standard concentration, *C*°, which is equal to 1 mol L^–1^ and can be effectively omitted. In turn, the stoichiometric concentration of phenol, *C*_0_, assuming that only aggregates up to tetramers exist, is given by *C*_0_ = ∑_*n* = 1_^4^ *nC*_*n*_. This mass-conservation condition, together with all three self-association equilibria, eq 6, completes a system of four equations whose solution provides the concentration of the monomer and the three aggregates.

### 2.3 Classical/Dynamic (**C/d**) Approach

The **C/d** approach leverages the sampling provided by an MD simulation (details of the simulation are given in Section 3.3). Our novel methodology determines aggregate populations directly comparable to the **Q/s** approach by identifying the same interaction pattern conformers also using the aggregate tool.

Namely, the proposed protocol provides the number of conformational pattern conformers at each step, *N*_*n*α*j*_, from the block-diagonalization adjacency matrix and subsequent classification of the aggregate. The equilibrium concentrations are then computed according to the following equation

7 $$\left\langle C_{n\alpha }\right\rangle =\frac{1}{N_{\mathrm{steps}}}\sum_{j}^{N_{\mathrm{steps}}}\frac{N_{n\alpha j}}{V_{j}}\frac{1}{N_{A}}$$

The population of the α-th interaction pattern is then given by

8 $$P_{n\alpha }^{C/d}=\frac{\left\langle C_{n\alpha }\right\rangle }{\sum_{\alpha }\left\langle C_{n\alpha }\right\rangle }$$

The global concentration of each aggregate is, in this case, given by the sum over all of the conformers of the same size, ⟨*C*_*n*_⟩ = ∑_α_⟨*C*_*n*α_⟩. Naturally, during the simulation, aggregation states larger than those of tetramers are also encountered, which will make the sum from the monomer to the tetramer lower than the stoichiometric phenol concentration, *C*_0_. However, as their fraction in the mixture is below 5%, they can be safely disregarded, therefore keeping the same monomer–dimer–trimer–tetramer equilibrium model as in the **Q/s** case. In this sense, to maintain consistency with the **Q/s** protocol, the equilibrium concentrations considered here are not those measured directly from the MD simulation but rather those obtained from the mass-conservation law (assuming species up to the tetramer) and the self-association equilibria, as shown in eq 6. The required equilibrium constants are obtained directly from the ratio of the average equilibrium concentrations of the aggregates within the simulation box

9 $$K_{n}^{C/d}=\frac{\langle C_{n}\rangle {\left(C_{0}\right)}^{n-1}}{{\left\langle C_{1}\right\rangle }^{n}}$$

In Table S8 of the SI, we report the concentrations computed directly from the MD simulations and those eventually used in this work. Although the overall trends are consistent, the MD populations reveal the noticeable presence of higher-order aggregates. In the most concentrated solution, these account for up to 15% of the total phenol population. Nevertheless, the molar concentrations of these larger species remain a small fraction of all aggregates in solution. Indeed, our results suggest that beyond the tetramer, the influence of higher-order aggregates is quite limited. Since the focus of this study is on comparing the **Q/s** and **C/d** approaches, we have decided to maintain our simplified treatment, including only aggregates up to the tetramer. We acknowledge, however, that the presence of higher-order aggregates raises certain limitations, which could be addressed in future work by adopting generalized methodologies that randomly select individual phenol molecules and explicitly consider their surrounding environments.

### 2.4 Summary of the Proposed Methodologies

The two strategies described above are summarized in the diagram presented in Figure 2. On the one hand, the **C/d** approach (top) applies the aggregate program to the snapshots generated along the MD trajectory. These snapshots typically contain aggregates of varying sizes, each spanning multiple interaction patterns. The aggregate program identifies the size and interaction pattern of each aggregate, ultimately providing their populations. On the other hand, the **Q/s** approach (bottom) implies an initial systematic conformational search that generates isomers for aggregates of different sizes. The aggregate program is subsequently run over these isomers to map them to their corresponding interaction-pattern conformers, and their populations are determined by accounting for the free energies of all of the isomers contributing to each interaction pattern.

**Figure 2:**
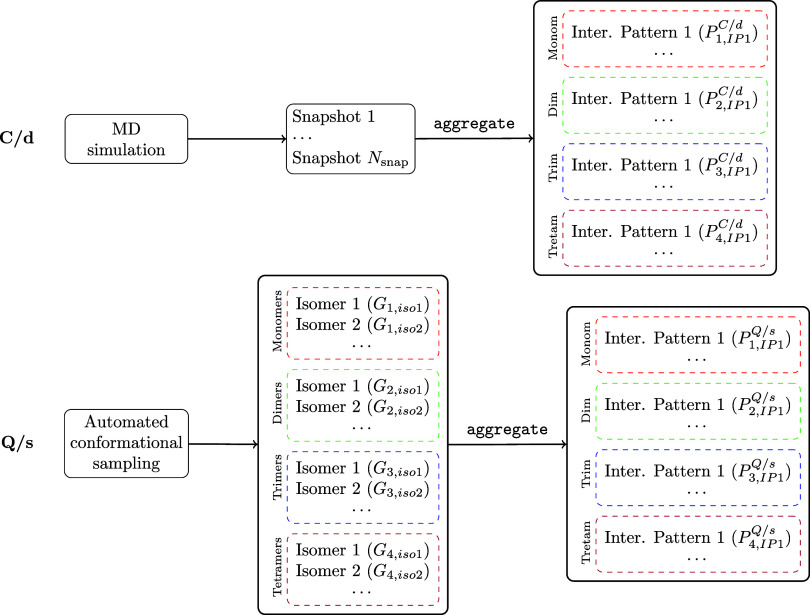
Diagram of the two protocols adopted in this work to obtain the populations of the different interaction-pattern conformers using **C/d** (above) and **Q/s** (below) strategies.

It is important to emphasize that both approaches yield populations for the same set of interaction-pattern conformers, as identified by the aggregate program. This ensures a consistent and reliable comparison between the populations derived from MD simulations (**C/d**) and those obtained via systematic conformational searches (**Q/s**).

Additionally, to maintain consistency, in all cases, we provide the populations for each interaction pattern corresponding to a specific aggregate size (up to tetramers). Then, the relative concentrations of monomers, dimers, trimers, and tetramers are computed from the equilibrium constants for aggregate formation. These constants are determined from either free energies (**Q/s** method, eq 5) or average populations (**C/d** method, eq 9).

### 2.5 Calculation of Averaged IR Spectra

Once the populations of the interaction-pattern conformers are obtained using both methodologies, their performance is assessed by evaluating the averaged broad IR O–H stretching band arising from the contributions of these conformers. A key step in this process is computing the IR spectra associated with each interaction-pattern conformer.

In this work, the IR spectra for each conformer are derived from the **Q/s** approach, assuming that the flexibility within the interaction pattern is properly captured by this methodology. Specifically, the spectra are obtained by averaging the harmonic IR spectra of all isomers mapping to a given interaction-pattern conformer. Vibrational frequencies were scaled using the scaling factor computed in this work (0.96) to correct for errors stemming from anharmonicity and the inherent approximations of the DFT functional. Further details on the calculation of the scaling factor are provided in Section C of the SI. The Boltzmann-averaged molar absorption coefficient for the conformer α, of size *n*, ε_*n*α_(ω), is computed as

10 $${\mathrm{\varepsilon ̅}}_{n\alpha }\left(\omega \right)=\sum_{i}w_{n\alpha i}\varepsilon_{n\alpha i}\left(\omega \right)$$

where *i* runs over all isomers contributing to the interaction pattern, and the relative weight of each isomer, *w*_*n*α*i*_, is given by

11 $$w_{n\alpha i}=\frac{e^{-\left(G_{n\alpha i}/\mathrm{RT}\right)}}{\sum_{i}e^{-\left(G_{n\alpha i}/\mathrm{RT}\right)}}$$

These molar absorption coefficients are then used to compute the total absorbance of the sample by exploiting the additivity of the Beer–Lambert law. The total absorbance is the sum of contributions from all conformers, weighted by their populations and macroscopic concentrations. The absorbance spectrum is expressed as

12 $$A\left(\omega \right)=l\sum_{n}^{4}C_{n}\sum_{\alpha }^{N_{\mathrm{IP}}^{\left(n\right)}}P_{n\alpha }{\mathrm{\varepsilon ̅}}_{n\alpha }\left(\omega \right)$$

Here, *l* is the cuvette path length, set to the experimental value;^68^ *C*_*n*_ is the concentration of the *n*-th aggregate, related to the stoichiometric concentration via the self-association equilibria (eq 6), which accounts from the monomer (*n* = 1) to the tetramer (*n* = 4), and *N*_IP_^(*n*)^ represents the number of interaction-pattern conformers for each aggregate size. *P_nα_* is the population of the conformer evaluated with either **Q/s** or **C/d** methods. This approach enables a quantitative comparison to experimental data at different concentrations. To facilitate this comparison, the computed absorbance spectra are converted into transmittance to match the experimental format.

The individual IR spectra of conformers are convoluted using a Lorentzian function with a half-width at half-maximum (HWHM) of 10 cm^–1^ for frequencies below 3200 cm^–1^ and above 3650 cm^–1^, a commonly adopted value. However, for frequencies in the 3200–3650 cm^–1^ range, corresponding to the O–H stretching of phenol aggregates, a Gaussian function with a larger HWHM of 60 cm^–1^ is applied. This increased broadening accounts for the inhomogeneous effects caused by the complex conformational flexibility of the aggregates. Frequencies below 3200 cm^–1^ correspond to C–H stretching vibrations, while those above 3650 cm^–1^ arise from free O–H stretching in bare phenol and chain- or branched-like aggregates. The large Gaussian broadening in the O–H region reflects the structural diversity of the aggregates, which induces significant inhomogeneous broadening.

## 3 Computational Details

### 3.1 Electronic Structure Calculations

All electronic structure calculations were performed with Gaussian^95^ using the B3LYP functional including D3 Grimme’s dispersion correction for improved noncovalent interactions. Geometry optimizations were carried out using the N07D^96^ basis set, including the environment effect within the IEF-PCM^85^ implicit solvation model. Thermal corrections are computed from the geometries and frequencies obtained at this level of theory. Final energies are refined by increasing the basis set size (N07Tdiff), computing the electronic gas phase and solvation-free energy contributions in eq 2 independently. Moreover, the basis set superposition error was corrected using the counterpoise correction in the calculation of the total gas-phase energies. For the standard state correction in eq 2, we used the value of the experimental density of CCl_4_.

### 3.2 Systematic Conformational Search

The PES of noncovalent complexes is characterized by the presence of many local, low-lying energy minima, which need to be taken into account effectively, as an insufficient conformational sampling could easily contribute several kcal mol^–1^ to the free-energy differences.^78^ In this work, we have conducted such conformational sampling with *AutoMeKin* package,^75^ with some modifications to ensure an efficient and complete as possible search. We note that a reliable screening is essential to reduce as much as possible the computation of vibrational frequencies for the clusters, which consume most of the computational time and represent by far the computational bottleneck of the entire procedure. The specific strategy employed to minimize the computational cost of conformational exploration is described in detail in the SI (Section B).

Of course, this modeling strategy based on partial optimizations can be coupled to other automated conformational search methods from the very beginning,^9,86,87^ or even MD simulations to obtain the candidate structures. In fact, after analyzing the MD trajectories, some interaction pattern conformers listed in Table 1 could not be identified using the procedure described above. To address this, we conducted a second round of conformational sampling *post hoc*, starting from a set of **C/d** structures classified as the missing conformers. Still, some isomers could not be optimized, but they can be safely ignored, because their low populations make their overall contribution to the spectrum negligible. The effect of expanding the aggregate set on the **Q/s** populations and the computed IR spectra is discussed in the SI (see Section E).

### 3.3 Molecular Dynamics Settings

The **C/d** approach involves unbiased MD simulations of phenol in carbon tetrachloride mixtures that were conducted with Gromacs 5.0.7.^97^ The simulation box contains a mixture of both compounds, with approximately 11,000 molecules in total. Specifically, we conducted MD simulations of three solutions with increasing solute concentrations (0.115, 0.45, and 1.1 M) incorporating 10,000 solvent molecules and 111, 434, and 1060 solute molecules into a starting cubic simulation box with edges of 12.0, 12.5, and 13.0 nm, respectively.

Over the simulation, bonds containing hydrogen atoms were constrained using the LINCS algorithm, allowing a time step of 1 fs for the integration of the equations of motion (using the leapfrog algorithm). Particle-mesh Ewald was considered for the calculation of long-range electrostatic forces using a cutoff of 1.1 nm. The same cutoff was used for the short-range van der Waals forces. The general AMBER force field (GAFF) was used to model solvent and solute molecules. GAFF parameters were taken directly from virtualchemistry.org.

After constructing and minimizing the simulation box, the system is equilibrated by subsequent 2 ns of *NVT* simulation (using the modified Berendsen–velocity rescale–thermostat with a time constant for coupling of 0.1 ps to keep the temperature constant at 298.15 K) followed by 15 ns of *NpT* simulation. Production trajectories were then extended for a total of 100 ns, with an *NpT* ensemble at 298 K and 1 atm. The Nose–Hoover thermostat and Parrinello–Rahman barostat were used in the *NpT* simulations with relaxation times of 1.0 and 5.0 ps, respectively. Configurations used in the subsequent analysis were extracted from the MD trajectory every 1 ps. Such an export rate was assessed by comparing the populations derived from a larger export frequency of 5 ps. The results obtained with both export frequencies show no significant differences beyond statistical uncertainty, thus validating our setup. The corresponding concentrations calculated using both snapshot frequencies are provided in Table S8 of the Supporting Information.

## 4 Results and Discussion

In this work, we have deployed two alternative strategies to account for the populations of different phenol aggregates, i.e., the **Q/s** and **C/d** methodologies described in Sections 2.2 and 2.3. In the following sections, we first introduce and compare the populations of aggregates provided by each of them and eventually use them to compute the average IR spectra, focusing on the O–H stretching region.

### 4.1 Conformational Sampling and Populations

The graph theory formalism adopted to identify the configurations of the aggregates provides a unified framework for analyzing the populations computed with both classical (**C/d**) and quantum (**Q/s**) methodologies. Namely, we characterize the different molecular configurations of aggregates based on connectivity patterns, allowing us to systematically classify aggregates of varying sizes (monomers, dimers, trimers, and tetramers), each with distinct interaction pattern conformers. These conformers, in turn, lead to different minima (isomers) that arise from the molecular flexibility.

In the **Q/s** approach, automated sampling generates many of these isomers, with all of them (and potentially more) appearing during MD sampling. However, tracking every possible isomer from the trajectory is infeasible. Instead, by using interaction-pattern conformers, which will generally encompass various minima in the **Q/s** approach and multiple configurations in the **C/d** MD sampling, we establish a common descriptor to classify configurations and enable a meaningful comparison of their populations with both methodologies.

As described in detail in the previous section, in the **Q/s** approach, the population of each conformer is derived from quantum mechanical partition functions, assuming a harmonic model around each minimum and summing the populations of all minima associated with the corresponding conformer. Conversely, in the **C/d** approach, the population is determined by counting the occurrences of the different conformers, as identified through MD sampling.

The population of aggregates with a given size can then be determined by summing the populations of all of the conformers corresponding to each aggregate. This enables us to determine the concentrations from the monomer to the tetramer, which can then be compared with experimental results, offering an initial assessment of the performance of both **Q/s** and **C/d** approaches. Our findings are summarized in Figure 3, which presents the concentrations of monomers and aggregates (up to tetramers) in terms of the stoichiometric concentration of phenol. Notably, the **Q/s** approach provides a continuous curve representing populations across concentrations, while the **C/d** approach is limited to the discrete systems simulated.

**Table 1:** Populations of the Different Conformers Computed with QM and Classical MD Methodologies

	dimer^a^	
	**C/d**	**Q/s**
OH-bonded	68.65	51.00
π–π	8.58	33.43
OH-π	22.76	15.56

	trimer^b^	
	**C/d**	**Q/s**
dimer-like	41.42	55.78
linear	48.67	23.82
cyclic	1.40	17.79
π–π bonded	4.13	2.55
OH–π bonded	4.41	

	tetramer^b^	
	**C/d**	**Q/s**
dimer-like	15.41	0.18
double dimer	10.59	3.67
trimer-like linear	26.80	5.24
trimer-like cycle	0.47	0.11
star	0.38	0.04
linear	34.53	32.32
cocktail	0.09	3.51
cyclic	9.68	54.96
π–π bonded	1.30	0.01
OH–π bonded	0.75	

a Computed averaging of the populations of dimer conformations of every MD simulation.

b Computed averaging of the populations of the aggregate conformations of the two MD simulations at higher concentration.

**Figure 3:**
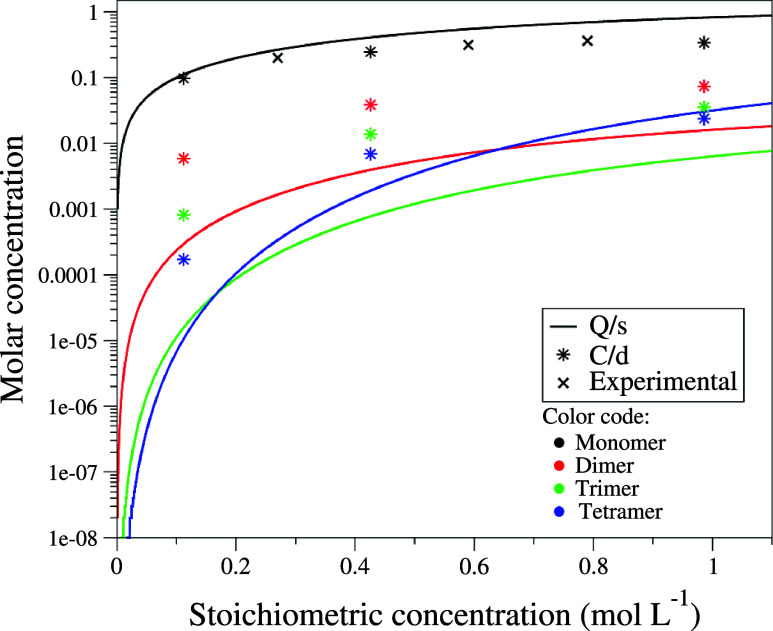
Overall population of aggregates (up to tetramers) with respect to phenol concentration, computed with **Q/s** (solid continuous lines) and **C/d** approach (stars). The experimental populations for the monomer from Schaefer et al.^66^ are included for reference (crosses).

Our results clearly highlight the different performances of the methodologies. In both cases, the monomer is the most populated species; however, the relative populations of the different aggregates differ between the two strategies. Specifically, in the **C/d** approach, aggregate populations follow the order dimer > trimer > tetramer over the entire range of concentrations. In the **Q/s** approach, this order holds only at low concentrations, but the tetramer population increases more sharply, surpassing the trimer population at concentrations above approximately 0.1 M and the dimer population at concentrations above approximately 0.5 M. Although no specific evidence on the relative populations of the aggregates is experimentally available, the monomer population derived from experiments^66^ aligns more closely with the results from the **C/d** method. Experimental estimations are available,^59–71^ however, for the self-association equilibrium constants, which can be compared with those computed with **Q/s** and **C/d**. Notably, equilibrium constants calculated with the **C/d** approach align better with the experimental values. In contrast, those derived from **Q/s** consistently shift the self-association equilibria toward the monomer compared to experimental findings. This explains the overpopulation of the monomer reported in Figure 3 with **Q/s**. The equilibrium constants used to generate the distribution curves shown in Figure 3, along with their experimental counterparts, are provided in Table S6 of the SI. Moreover, to facilitate the analysis, in Table S7 we also provide the equilibrium constants referred to reactions normalized to a stoichiometric coefficient of one for the monomer, which show a steady increase with aggregate size. The relative values of the equilibrium constants between both approaches also explain the observed trends for each method reported in Figure 3. Namely, the ratio between the **C/d** and **Q/s** equilibrium constants decreases as aggregate size increases: from 4.8 for the dimer to 3.9 for the trimer and down to 2.1 for the tetramer. This trend leads to a relative enhancement of the tetramer’s population in the **Q/s** approach compared to that of the smaller aggregates. As a result, the tetramer becomes the dominant species in the **Q/s** predictions at much lower concentrations compared to the **C/d** ones. For example, Figure 3 shows that the tetramer surpasses the trimer at approximately 0.2 mol L^–1^, and the dimer surpasses the trimer at around 0.6 mol L^–1^. In contrast, with the **C/d** approach, although the population of the tetramer increases and approaches those of the trimer and dimer, it does not surpass those within the concentration range considered.

We now turn our attention to the relative distribution of interaction-pattern conformers for each aggregate. Table 1 lists all such interaction patterns in the first column, along with the populations of each species obtained using both the **Q/s** and **C/d** approaches, allowing for a more detailed comparison of the performance of each strategy.

For dimers, the populations of different conformers are relatively similar across both methods, with the hydrogen bonded conformer being the most populated in both cases. However, notable differences emerge as the aggregate size increases. For trimers, the most populated conformer changes from dimer-like with **Q/s** to linear with **C/d**. This trend, favoring linear conformers in the **C/d** approach, continues with tetramers, where linear conformers remain the most populated with **C/d**, whereas the cyclic conformer becomes dominant with **Q/s**.

Notably, the cyclic conformer represents one of the most striking differences between the two methodologies: it is significantly populated in both trimer and tetramer aggregates with **Q/s**, while **C/d** predicts a relatively low population. This high population of cyclic structures observed with **Q/s** may, in fact, be overestimated, as previously noted in similar studies.^52^ The relative accuracy of each strategy will be evaluated further in the next section, where these populations are used to simulate the broad IR O–H stretching band of phenol as a function of concentration.

### 4.2 Simulation of the IR Spectrum

We focused on simulating the OH region of the IR spectra for the mixtures. The spectral shapes in this region arise from the variety of aggregates formed, each exhibiting distinct IR features whose populations depend on the relative concentrations of phenol and carbon tetrachloride, as shown in the previous section. This makes calculating this spectral region particularly challenging, as it requires careful consideration of the interplay of nonbonding interactions, especially hydrogen bonding, that influence the relative populations of the aggregates.^52^ For this reason, such a simulation serves as a valuable benchmark to assess the accuracy of the populations computed using the two methodologies proposed in this work, **Q/s** and **C/d**.

To isolate the effects of relative populations, we evaluated the spectra from a weighted average over the interaction-pattern conformers, using identical individual spectra for each of them. In turn, these individual spectra are computed as weighted averages of the IR spectra for each optimized isomer, calculated at the DFT level, with relative weights provided by the **Q/s** method. The spectra for each conformer are included in Section F of the SI.

Let us begin by analyzing the shape and position of the different aggregates displayed in Figure 4 for both the **Q/s** and **C/d** strategies. In general, we find the expected behavior,^58,88–90^ with the position of the band going toward lower frequencies as the size of the aggregate increases. It should be noted, however, that the average bands for each aggregate generally consist of a complex superposition of different conformers. In this sense, since not all conformers are solely dictated by hydrogen bonds, e.g., those with π-π stacking interactions, there are some features that lie at higher frequencies. Moreover, it is worth noting that the shape of each aggregate also depends on the adopted strategy. Namely, **C/d** generally results in broader spectra, suggesting the inclusion of a wider variety of species in the average, which follows the distribution of relative populations displayed in Table 1.

**Figure 4:**
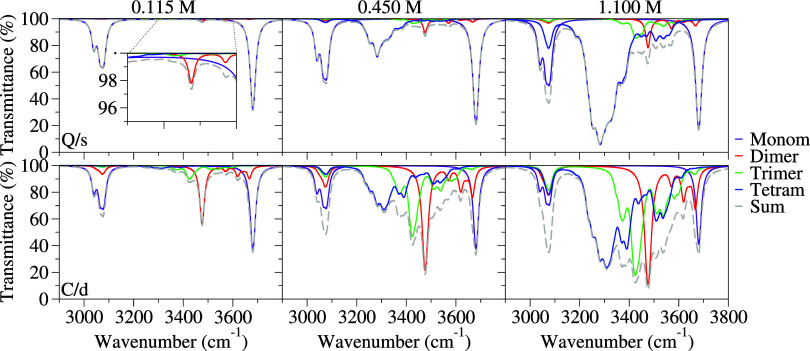
IR spectrum of phenol–tetrachloride mixtures at different concentrations, simulated through **C/d** (bottom panels) and **Q/s** (top panels) strategies. The contributions of monomers to tetramers are depicted individually. Inset: zoom of the plot to make evident the small bands appearing in the intermediate region in the case of a concentration of 0.115 M with the **Q/s** approach.

We continue by identifying the contributions of monomers, dimers, trimers, and tetramers to the final spectra. It is noteworthy that the spectra for each aggregate differ not only in intensity between methods but also in shape. The relative weight of each aggregate aligns with the populations shown in Figure 3. For example, at the lowest concentration, the spectrum is clearly dominated by the monomer in the **Q/s** method, while the contribution of the dimer, although lower than that of the monomer, is still notable in the **C/d** approach.

As the phenol concentration increases to 0.450 M, **Q/s** reveals a predominant contribution of the tetramer, even if the monomer remains as the major component. In contrast, **C/d** shows a more balanced contribution across the different aggregates. Finally, in the most concentrated sample, the tetramer band is the most intense with **Q/s**, while **C/d** again provides a more evenly distributed contribution among the aggregates with an increased contribution of the tetramer compared to lower concentrations.

The spectra in Figure 4 display a complex band structure with numerous well-resolved peaks in the 3150–3600 cm^–1^ region. This effect, observed with both the **Q/s** and especially the **C/d** strategies, contrasts with the broad, structureless band seen in experimental data.^68^ This discrepancy likely arises from the conformational flexibility associated with a relatively flat potential surface and the sensitivity of OH stretching frequencies to hydrogen bonding in this region. Accurately capturing this effect would require incorporating contributions from out-of-equilibrium structures, which, while accessible through the **C/d** protocol, lies beyond the scope of this study. Instead, we address this phenomenon phenomenologically by applying larger Gaussian broadening specifically in this region to account for inhomogeneous broadening. This adjustment results in smoother curves that more closely resemble the experimental band. Specifically, we apply Gaussian broadening with a half-width at half-maximum (HWHM) of 60 cm^–1^ in the 3150–3600 cm^–1^ region while retaining a Lorentzian broadening with HWHM of 10 cm^–1^ in the rest of the spectral region.

The resulting spectra, simulated with both **Q/s** and **C/d**, alongside the experimental one,^68^ are shown in Figure 5. Focusing first on the experimental data, it is evident that for all concentrations there are a number of bands that remain nearly unchanged. These can be associated with the monomer, at approximately 3600 cm^–1^, and CH stretching vibrations (the double-peaked band around 3000 cm^–1^). Both of these features are identifiable in the simulated spectra with the **Q/s** and **C/d** methods, with slight deviations in position that can be attributed to the usual inaccuracies in DFT-computed frequencies at the harmonic level even if using a scale factor.

**Figure 5:**
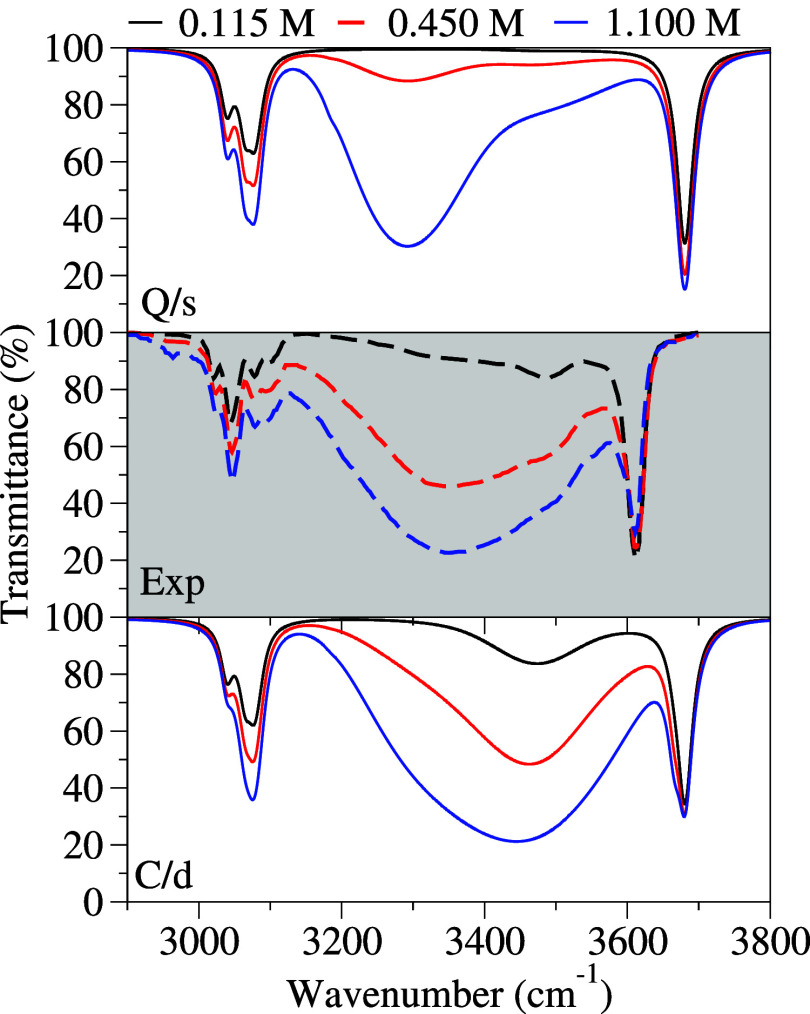
IR spectrum of phenol-tetrachloride mixtures at different concentrations (0.115, 0.450, and 1.100 M), simulated through **C/d** (bottom panel) and **Q/s** (top panel) strategies, with a phenomenological broadening in the O–H region using a Gaussian with a HWHM of 60 cm^–1^. The experimental spectra for these concentrations are included in the central panel.

The main concentration-dependent changes in the spectrum occur in the intermediate region between these relatively stable bands. Signals in this region correspond to the aggregates as discussed above, and in the following, we will focus our discussion on them, comparing the experiments with the theoretical simulations at each concentration.

Starting with the most diluted sample, the experimental spectrum shows a band in the intermediate region located just below the band of the monomer. While appreciable, this band is significantly less intense than the monomer one. In the simulations, the **Q/s** method does not produce any significant signal in this region, whereas **C/d** displays a small contribution from the dimer, which is consistent with the experimental data, although shifted to lower frequencies.

Moving to the intermediate concentration, the experimental band in the intermediate region broadens and increases in intensity. In this case, the **Q/s** approach produces a signal mainly associated with the tetramer, which is again notably less intense than the experimental band. In contrast, the **C/d** method is able to capture the broadening and intensity increase in this intermediate region, resulting in a simulated band that aligns quite well with the experimental data, although it is shifted to higher frequencies, suggesting a possible underestimation of tetramer-related bands.

Finally, for the most concentrated sample, the experimental band in the intermediate region shows a moderate increase in intensity compared to that of the previous concentration, but its shape remains mostly the same, with a slight enhancement on the low-frequency side. The **Q/s** method displays a significant increase in intensity, dominated by the tetramer-related band. Although the intensity is similar to that in the experiment, the band is narrower and overly centered on the tetramer. In contrast, the **C/d** method provides a simulated band that follows the experimental trend, showing an increase in intensity compared to the previous concentration. Regarding the shape, there is a change induced by the increased contribution of the tetramer, resulting in a band that aligns more closely with the experimental data.

Following analysis of the OH region in the IR spectrum (Figure 5), it is evident that the **C/d** strategy outperforms the **Q/s** approach, indicating that the populations derived from the classical MD trajectory better reflect the experimental behavior. But which factors account for the superiority of **C/d** populations over those from **Q/s**? To address this question, we need to consider the two thermodynamic contributions to population distributions: the enthalpic and entropic terms associated with the formation of aggregate.

The enthalpic contribution is primarily dictated by intermolecular interactions, including hydrogen bonds and π-π interactions. In this sense, it is expected that the dispersion-corrected DFT calculations in the **Q/s** approach will outperform the force field calculations applied in **C/d**. Regarding the treatment of the environment, **Q/s** employs an implicit solvation model (PCM), which although may face limitations in describing specific interactions such as hydrogen bonds, provides a reasonable account of polarization effects, which are likely more relevant in the case of carbon tetrachloride solutions. Indeed, the potential inaccuracies in the FF, which, despite offering an explicit description of the solvent, completely neglects key effects such as polarization, could partly explain some of the deficiencies in **C/d**, such as its apparent underestimation of tetramer populations. Therefore, enthalpy differences cannot explain the better performance of the **C/d** strategy. The key to understanding this difference would necessarily lie in the entropic term, which is linked to the vast number of configurations arising from the significant molecular flexibility in loosely bound aggregates. This implies that many configurations are necessary to achieve population distributions in the experimental system, which becomes unmanageable for the automated conformational search of the **Q/s** approach. Conversely, this conformational flexibility is, by construction, effectively captured by the MD simulation adopted in the **C/d** strategy.

The entropy-based analysis further elucidates the prevalence of cyclic conformers in the **Q/s** approach, whereas linear conformers are more common with the **C/d** strategy, as shown in Table 1. The significantly larger number of configurations available to linear conformers makes it challenging to identify all possible configurations via the automated search used in the **Q/s** approach. This limitation leads to an underestimation of the entropic contributions that favor linear conformers over cyclic conformers, which, while being enthalpically favorable, display a much lower entropy. Thus, the apparent overestimation of cyclic structures with **Q/s**-like approaches previously reported,^52^ would rather correspond to an underestimation of chain-like configurations.

Such an analysis in terms of entropy also helps rationalize the trends observed in the equilibrium constants for aggregation discussed in relation to the populations in Figure 3. While the constants are consistently lower with the **Q/s** approach, they tend to converge toward the **C/d** values as aggregate size increases. This can be understood by considering the balance between enthalpic and entropic contributions to the free energy: as the aggregate size grows, the number of stabilizing interactions (e.g., hydrogen bonds or π-stacking) increases, making the enthalpic term, similarly described in both approaches, more dominant. Since the entropic term, which is poorly captured in **Q/s**, becomes less relevant for larger aggregates, the agreement between **Q/s** and **C/d** improves with aggregate size.

It is important to note that once an optimized configuration of an isomer is identified in the **Q/s** method, conformational motions around that configuration are included through the partition function, which is based on harmonic model surfaces and a quantum mechanical treatment of vibrations. For intramolecular vibrations, the entropic terms are better described by this quantum description compared to the classical treatment of MD trajectories in the **C/d** approach. However, aggregate formation is primarily governed by intermolecular nonbonded interactions, which lead to loose nuclear motions that are effectively described at the classical level. For the study of nonbonded species, the **C/d** strategy thus emerges as a very suitable approach.

Moreover, the **C/d** approach demonstrates remarkably good agreement with experimental data. This is a notable achievement, as the shape of the OH band results from a complex interplay of nonbonding interactions governing aggregate formation, which in turn defines the band structure.

## 5 Conclusions

In this study, we have introduced and evaluated two distinct methodologies for sampling the nuclear degrees of freedom in a system governed primarily by nonbonded interactions: a liquid mixture of phenol and carbon tetrachloride. Our aim was to determine which protocol provides a more reliable description of the aggregate populations. Subsequently, we assess their performance by comparing the average IR spectra, computed with the populations resulting from each methodology, against the experimental one at varying concentrations, particularly focusing on the OH stretching region.

The first method employs a systematic conformational search followed by a potential energy surface (PES) expansion around each identified structure. This approach, which we termed **Q/s**, computes the nuclear partition function by assuming a harmonic PES and treats vibrational modes at a quantum mechanical level (with rotational and translational contributions considered semiclassically and classically, respectively). The second method, termed **C/d**, calculates populations by conducting a classical molecular dynamics (MD) simulation, thus treating nuclear motion fully classically.

A rigorous framework was essential to identify and categorize aggregates consistently across both methodologies. We accomplished this through a graph-theory classification based on nonbonded interaction patterns that define each aggregate, including hydrogen bonds, π-π stacking, and π–OH bonds. The **Q/s** approach identifies several optimized structures for each conformer during the automated search, capturing some degree of conformational flexibility. The **C/d** approach inherently includes this flexibility through MD trajectories.

Upon comparing the aggregate populations obtained with the **Q/s** and **C/d** approaches, we found that they align closely for smaller aggregates, such as dimers, but diverge significantly as the aggregate size increases. Specifically, **Q/s** tends to favor cyclic conformers, while **C/d** predicts a predominance of linear aggregates. This divergence arises due to the different entropic and enthalpic contributions captured by each method: while **Q/s** benefits from quantum mechanical accuracy to evaluate the interactions, it under-represents the vast configurational entropy associated with loosely bound aggregates due to the limitations of systematic conformational search. In contrast, **C/d** captures a more realistic entropic profile by exploring a broader ensemble of conformers through MD, even though it approximates intermolecular interactions by using a molecular mechanics force field.

The ability of the **Q/s** method to align with experimental observations is limited by the extent of its conformational search. While an exhaustive search could yield results closer to those of **C/d**, nonbonded aggregates exhibit high configurational entropy, imposing practical limits on systematic search-based approaches. Aggregates with such loosely bound, flexible structures inherently challenge even sophisticated automated search techniques as capturing their full entropy becomes infeasible. Our findings thus indicate that the **C/d** approach provides a more reliable representation of aggregate populations in this system, as demonstrated by its superior performance in simulating IR spectral regions sensitive to nonbonded interactions, particularly, the broad OH stretching band.

To achieve such complete conformational sampling within **Q/s**-based strategies, we recommend carrying out the screening of the structures not solely based on energy criteria (relative population of the aggregates) but on entropy-like criteria based on the convergence of the multiconformational contributions to the free-energy, or equivalently, the conformational entropy.^91^ Moreover, when possible, this convergence must be achieved for each isomer group. Naturally, this is associated with a significant increase in computational time, particularly for noncovalent complexes, where large basis sets or the counterpoise correction are required to mitigate basis set superposition error.

It is noteworthy that the **C/d** approach not only outperforms **Q/s** but also achieves an impressive absolute accuracy. This method closely reproduces the experimental trends of the broad O–H stretching band in IR as phenol concentration changes. This is indeed a challenging task, given that the observed broadband reflects the complex network of nonbonding interactions that give rise to different populations of conformers as the concentrations increase. With minor deviations, **C/d** captures these experimental variations nearly quantitatively. In essence, its capacity to handle loosely bound, entropically driven conformers makes the **C/d** approach a compelling choice for systems governed by weak, noncovalent interactions. Of course, it is also possible to adapt **Q/s** approaches to incorporate the entropic effects in an effective manner, e.g., through Bayesian inference.^52^ In this context, our results can also be helpful in improving such methodologies.

The observed deviations with the **C/d** approach are likely due, in part, to inaccuracies in the FF used to evaluate the intermolecular interactions. In this sense, this system may serve as an effective benchmark to assess refined intermolecular FF quality. In this study, we adopted standard GAFF parameters for the intermolecular potential, though we are currently pursuing refined parametrizations based on quantum mechanical (QM) calculations, as implemented in Picky.^7,92^ These adjustments may further improve the agreement with experimental data. Moreover, another source of potential inaccuracies arises from the presence of aggregates larger than the tetramer, whose influence increases with the concentration. One route for improving the simulations involves extending the **C/d** approach to avoid strict reliance on predefined interaction patterns, e.g., by randomly selecting individual phenol molecules and subsequently incorporating their environment in the analysis. This strategy is currently being investigated by our group.

Another limitation is that the method does not directly account for the inhomogeneous broadening that affects the region where aggregate signals emerge. In this work, we included this effect phenomenologically, adding specific Gaussian broadening to this region to mimic the experimental band closely. An alternative to incorporate such effects from first principles could involve mixed quantum-classical strategies similar to those used in electronic spectroscopy,^93^ where the QM spectra of stiff solute degrees of freedom are computed across configurations generated via MD simulations. For IR spectra, this would require spectral frequency calculations at nonstationary points, which is feasible today using curvilinear internal coordinates.^94^ These strategies are currently being explored to achieve a fully ab initio simulation of the challenging broad OH IR band.

## Supplementary Material



## Notes

Comprehensive explanation of the thresholds used to define noncovalent interactions and the interaction patterns employed to classify aggregate conformations; details on the determination of the scaling factors for harmonic frequencies used in this work; examination of the dependence of **C/d** populations and computed spectra on the thresholds and broadening parameter (HWHM), as well as the convergence of **Q/s** populations and both sets of spectra with the chosen QM set and HWHM parameter; concentration measurements from the MD simulations, contributions to the free energy of QM-optimized conformers; and a comparison of the calculated equilibrium constants with values reported in the literature (PDF)

## References

[1] Jena S., Dutta J., Tulsiyan K. D., Sahu A. K., Choudhury S. S., Biswal H. S. (2022). Noncovalent interactions in proteins and nucleic acids:
beyond hydrogen bonding and *π*-stacking. Chem. Soc. Rev..

[2] Grimme S. (2004). Accurate description
of van der Waals complexes by density functional theory including
empirical corrections. J. Comput. Chem..

[3] Antony J., Grimme S. (2006). Density functional
theory including dispersion corrections
for intermolecular interactions in a large benchmark set of biologically
relevant molecules. Phys. Chem. Chem. Phys..

[4] Tomasi J., Mennucci B., Cammi R. (2005). Quantum Mechanical
Continuum Solvation
Models. Chem. Rev..

[5] Liu C., Piquemal J.-P., Ren P. (2019). AMOEBA+ Classical
Potential for Modeling
Molecular Interactions. J. Chem. Theory Comput..

[6] Inakollu V. S., Geerke D. P., Rowley C. N., Yu H. (2020). Polarisable force fields:
what do they add in biomolecular simulations?. Curr. Opin. Struct. Biol..

[7] Prampolini G., Livotto P. R., Cacelli I. (2015). Accuracy of Quantum
Mechanically
Derived Force-Fields Parameterized from Dispersion-Corrected DFT Data:
The Benzene Dimer as a Prototype for Aromatic Interactions. J. Chem. Theory Comput..

[8] Ferro-Costas D., Fernández-Ramos A. (2023). New computational tools
for chemical
kinetics: the Cathedral Package. Theor. Chem.
Acc..

[9] Pracht P., Grimme S., Bannwarth C., Bohle F., Ehlert S., Feldmann G., Gorges J., Müller M., Neudecker T., Plett C., Spicher S., Steinbach P., Wesołowski P. A., Zeller F. (2024). CREST-A program for
the exploration
of low-energy molecular chemical space. J. Chem.
Phys..

[10] Kosztolányi T., Bakó I., Pálinkás G. (2003). Hydrogen bonding in
liquid methanol, methylamine, and methanethiol studied by molecular-dynamics
simulations. J. Chem. Phys..

[11] Hamad S., Hughes C. E., Catlow C. R. A., Harris K. D. M. (2008). Clustering of
Glycine Molecules in Aqueous Solution Studied by Molecular Dynamics
Simulation. J. Phys. Chem. B.

[12] Bernardes C. E. S. (2017). AGGREGATES:
Finding structures in simulation results of solutions. J. Comput. Chem..

[13] Choi S., Parameswaran S., Choi J.-H. (2020). Understanding alcohol aggregates
and the water hydrogen bond network towards miscibility in alcohol
solutions: graph theoretical analysis. Phys.
Chem. Chem. Phys..

[14] Saiz L., Padró J. A., Guàrdia E. (1997). Structure and Dynamics of Liquid
Ethanol. J. Phys. Chem. B.

[15] Gavezzotti A. (1999). Molecular
Aggregation of Acetic Acid in a Carbon Tetrachloride Solution: A Molecular
Dynamics Study with a View to Crystal Nucleation. Chem. - Eur. J..

[16] Khurana E., Nielsen S. O., Ensing B., Klein M. L. (2006). Self-Assembling
Cyclic Peptides: Molecular Dynamics Studies of Dimers in Polar and
Nonpolar Solvents. J. Phys. Chem. B.

[17] Chen J., Trout B. L. (2008). Computational Study
of Solvent Effects on the Molecular
Self-Assembly of Tetrolic Acid in Solution and Implications for the
Polymorph Formed from Crystallization. J. Phys.
Chem. B.

[18] Gaines E., Maisuria K., Di Tommaso D. (2016). The role of
solvent in the self-assembly
of m-aminobenzoic acid: a density functional theory and molecular
dynamics study. CrystEngComm.

[19] Gaines E., Di Tommaso D. (2018). Solvation
and Aggregation of Meta-Aminobenzoic Acid
in Water: Density Functional Theory and Molecular Dynamics Study. Pharmaceutics.

[20] Tanaka H., Nakanishi K., Touhara H. (1985). Computer experiments on aqueous solutions.
VII. Potential energy function for urea dimer and molecular dynamics
calculation of 8 mol % aqueous solution of urea. J. Chem. Phys..

[21] Hamad S., Moon C., Catlow C. R. A., Hulme A. T., Price S. L. (2006). Kinetic
Insights into the Role of the Solvent in the Polymorphism of 5-Fluorouracil
from Molecular Dynamics Simulations. J. Phys.
Chem. B.

[22] Chaiwongwattana S., Sagarik K. (2009). Structures
and dynamics of phenol clusters in benzene
solutions. Chem. Phys..

[23] Yani Y., Chow P. S., Tan R. B. H. (2012). Glycine
Open Dimers in Solution:
New Insights into *α*-Glycine Nucleation and
Growth. Cryst. Growth Des..

[24] Gavezzotti A., Filippini G., Kroon J., van Eijck B. P., Klewinghaus P. (1997). The Crystal
Polymorphism of Tetrolic Acid (CH3C CCOOH):
A Molecular Dynamics Study of Precursors in Solution, and a Crystal
Structure Generation. Chem. - Eur. J..

[25] Ramondo F., Bencivenni L., Caminiti R., Pieretti A., Gontrani L. (2007). Dimerisation
of urea in water solution: a quantum mechanical investigation. Phys. Chem. Chem. Phys..

[26] Barth, D. ; Bougueroua, S. ; Gaigeot, M.-P. ; Quessette, F. ; Spezia, R. ; Vial, S. A New Graph Algorithm for the Analysis of Conformational Dynamics of Molecules. In Lecture Notes in Electrical Engineering; Springer, 2016; Vol. 363, pp 319–326.

[27] Bougueroua S., Spezia R., Pezzotti S., Vial S., Quessette F., Barth D., Gaigeot M.-P. (2018). Graph theory for automatic structural
recognition in molecular dynamics simulations. J. Chem. Phys..

[28] Aida M., Akase D. (2019). Hydrogen-bond pattern
to characterize water network. Pure Appl. Chem..

[29] Leal A. M. M., Kulik D. A., Smith W. R., Saar M. O. (2017). An overview of computational
methods for chemical equilibrium and kinetic calculations for geochemical
and reactive transport modeling. Pure Appl.
Chem..

[30] Smith W. R., Qi W. (2018). Molecular Simulation of Chemical Reaction Equilibrium by Computationally
Efficient Free Energy Minimization. ACS Cent.
Sci..

[31] Noroozi J., Smith W. R. (2019). An Efficient Molecular Simulation Methodology for Chemical
Reaction Equilibria in Electrolyte Solutions: Application to CO2 Reactive
Absorption. J. Phys. Chem. A.

[32] Watanabe H., Iwata S. (1996). Theoretical studies
of geometric structures of phenol-water clusters
and their infrared absorption spectra in the O-H stretching region. J. Chem. Phys..

[33] Chaudret R., de Courcy B., Contreras-Garcıa J., Gloaguen E., Zehnacker-Rentien A., Mons M., Piquemal J.-P. (2014). Unraveling non-covalent
interactions within flexible biomolecules: from electron density topology
to gas phase spectroscopy. Phys. Chem. Chem.
Phys..

[34] Ludwig R., Reis O., Winter R., Weinhold F., Farrar T. C. (1998). Quantum
Cluster Equilibrium Theory of Liquids: Temperature Dependence of Hydrogen
Bonding in Liquid N-Methylacetamide Studied by IR Spectra. J. Phys. Chem. B.

[35] Blasius J., Kirchner B. (2020). Cluster-Weighting in Bulk Phase Vibrational Circular
Dichroism. J. Phys. Chem. B.

[36] Malloum A., Fifen J. J., Conradie J. (2020). Exploration
of the potential energy
surfaces of small ethanol clusters. Phys. Chem.
Chem. Phys..

[37] Malloum A., Fifen J. J., Conradie J. (2020). Theoretical
infrared spectrum of
the ethanol hexamer. Int. J. Quantum Chem..

[38] Kirchner B., Blasius J., Esser L., Reckien W. (2021). Predicting Vibrational
Spectroscopy for Flexible Molecules and Molecules with Non-Idle Environments. Adv. Theory Simul..

[39] Teh S., Hsu P.-J., Kuo J.-L. (2021). Size of
the hydrogen bond network
in liquid methanol: a quantum cluster equilibrium model with extensive
structure search. Phys. Chem. Chem. Phys..

[40] Malloum A., Dhaouadi Z., Conradie J. (2023). Quantum cluster
equilibrium prediction
of liquid ethanol. J. Mol. Liq..

[41] Malloum A., Conradie J. (2024). Structures of DMSO
clusters and quantum cluster equilibrium
(QCE). J. Mol. Graphics Modell..

[42] Maya J., Malloum A., Fifen J. J., Dhaouadi Z., Fouda H. P. E., Conradie J. (2024). Quantum cluster equilibrium
theory applied to liquid
ammonia. J. Comput. Chem..

[43] Perera A. S., Thomas J., Poopari M. R., Xu Y. (2016). The Clusters-in-a-Liquid
Approach for Solvation: New Insights from the Conformer Specific Gas
Phase Spectroscopy and Vibrational Optical Activity Spectroscopy. Front. Chem..

[44] Perera A. S., Cheramy J., Merten C., Thomas J., Xu Y. I. R. (2018). Raman,
and Vibrational Optical Activity Spectra of Methyl Glycidate in Chloroform
and Water: The Clusters-in-a-liquid Solvation Model. ChemPhysChem.

[45] Le
Barbu-Debus K., Scherrer A., Bouchet A., Sebastiani D., Vuilleumier R., Zehnacker A. (2018). Effect of puckering motion and hydrogen
bond formation on the vibrational circular dichroism spectrum of a
flexible molecule: the case of (S)-1-indanol. Phys. Chem. Chem. Phys..

[46] Le
Barbu-Debus K., Bowles J., Jähnigen S., Clavaguéra C., Calvo F., Vuilleumier R., Zehnacker A. (2020). Assessing cluster models of solvation for the description
of vibrational circular dichroism spectra: synergy between static
and dynamic approaches. Phys. Chem. Chem. Phys..

[47] Le
Barbu-Debus K., Zehnacker A. (2021). Competition between inter and intramolecular
hydrogen bond evidenced by vibrational circular dichroism spectroscopy:
The case of (1S,2R)-(−)-cis-1-amino-2-indanol. Chirality.

[48] Kutsyk A., Ilchenko O., Pilhun Y., Nikonova V., Obukhovsky V. (2022). Complex formation
in methanol-chloroform solutions: Vibrational spectroscopy and quantum
cluster equilibrium study. J. Mol. Liq..

[49] Yang Y., Cheramy J., Brehm M., Xu Y. (2022). Raman Optical Activity
of N-Acetyl-L-Cysteine in Water and in Methanol: The “Clusters-in-a-Liquid”
Model and ab Initio Molecular Dynamics Simulations. ChemPhysChem.

[50] Puente A. R., Polavarapu P. L. (2023). Influence of microsolvation on vibrational circular
dichroism spectra in dimethyl sulfoxide solvent: A Bottom-Up approach
using Quantum cluster growth. Spectrochim. Acta,
Part A.

[51] Perera A. S., Carlson C. D., Cheramy J., Xu Y. (2023). Infrared and vibrational
circular dichroism spectra of methyl *β*-D-glucopyranose
in water: The application of the quantum cluster growth and clusters-in-a-liquid
solvation models. Chirality.

[52] Blasius J., Drysch K., Pilz F. H., Frömbgen T., Kielb P., Kirchner B. (2023). Efficient Prediction
of Mole Fraction
Related Vibrational Frequency Shifts. J. Phys.
Chem. Lett..

[53] Weinhold F. (1998). Quantum cluster
equilibrium theory of liquids: General theory and computer implementation. J. Chem. Phys..

[54] Kirchner B., Spickermann C., Lehmann S. B., Perlt E., Langner J., von Domaros M., Reuther P., Uhlig F., Kohagen M., Brüssel M. (2011). What can clusters tell us about the bulk?: Peacemaker:
Extended quantum cluster equilibrium calculations. Comput. Phys. Commun..

[55] Brüssel M., Perlt E., von Domaros M., Brehm M., Kirchner B. (2012). A one-parameter
quantum cluster equilibrium approach. J. Chem.
Phys..

[56] von
Domaros M., Perlt E., Ingenmey J., Marchelli G., Kirchner B. (2018). Peacemaker2: Making clusters talk about binary mixtures
and neat liquids. SoftwareX.

[57] Frömbgen T., Drysch K., Zaby P., Dölz J., Ingenmey J., Kirchner B. (2024). Quantum Cluster Equilibrium
Theory
for Multicomponent Liquids. J. Chem. Theory
Comput..

[58] Laurence, C. ; Berthelot, M. ; Graton, J. PATAI’S Chemistry of Functional Groups; John Wiley & Sons, Ltd, 2009.

[59] Coggeshall N. D., Saier E. L. (1951). Infrared Absorption Study of Hydrogen Bonding Equilibria. J. Am. Chem. Soc..

[60] Huggins C. M., Pimentel G. C., Shoolery J. N. (1956). Proton Magnetic Resonance Studies
of the Hydrogen Bonding of Phenol, Substituted Phenols and Acetic
Acid. J. Phys. Chem. A.

[61] Saunders M., Hyne J. B. (1958). Study of hydrogen
bonding in systems of hydroxylic
compounds in carbon tetrachloride through the use of NMR. J. Chem. Phys..

[62] Maguire M. M., West R. (1961). Hydrogen-bonding studies VII. Near infrared spectroscopic studies
of the intermolecular hydrogen bonding of phenol, p-cresol and p-chlorophenol. Spectrochim. Acta.

[63] Singh S., Rao C. N. R. (1967). Spectroscopic studies of self-association due to hydrogen
bonding. J. Phys. Chem. A.

[64] Whetsel, K. B. ; Lady, J. H. Self-association of phenol in nonpolar solvents. In Spectrometry of Fuels; Springer, 1970; pp 259–249.

[65] Woolley E. M., Travers J. G., Erno B. P., Hepler L. G. (1971). Molecular
association
of hydrogen-bonding solutes. Phenol in carbon tetrachloride. J. Phys. Chem. A.

[66] Schaefer T., Rowbotham J. B., Chum K. (1976). The proton magnetic resonance spectra
of phenol in the absence of intermolecular proton exchange. Can. J. Chem..

[67] Lin L.-N., Christian S. D., Tucker E. E. (1978). Solute activity study of the self-association
of phenol in cyclohexane and carbon tetrachloride. J. Phys. Chem. A.

[68] Petelenz B. U., Shurvell H. (1980). Factor analysis as a complement to infrared band resolution.
VII. The temperature dependence of the self association of phenol
in carbon tetrachloride solution. Can. J. Chem..

[69] Josefiak C., Schneider G. (1980). Determination
of reaction volumes of hydrogen-bonding
equilibria by high-pressure near-infrared spectroscopy. 2. Self-association
of phenol in CCl4 up to 1 kbar. J. Phys. Chem.
A.

[70] Frohlich H. (1993). Using infrared
spectroscopy measurements to study intermolecular Hhydrogen bonding:
Calculating the degree of association, equilibrium constant, and bond
energy for hydrogen bonding in benzyl alcohol and phenol. J. Chem. Educ..

[71] Lessinger L. (1995). Hydrogen-bonding
equilibrium in phenol analyzed by NMR spectroscopy. J. Chem. Educ..

[72] Galvez, J. P. aggregate, a graph-theoretical tool to indentify interaction patterns in Molecular Dynamics simulations 2024. https://github.com/jpablogalvez/Aggregate.

[73] Martínez-Núñez E. (2015). An automated
method to find transition states using chemical dynamics simulations. J. Comput. Chem..

[74] Martínez-Núñez E. (2015). An automated
transition state search using classical trajectories initialized at
multiple minima. Phys. Chem. Chem. Phys..

[75] Martínez-Núñez E., Barnes G. L., Glowacki D. R., Kopec S., Peláez D., Rodrıguez A., Rodrıguez-Fernández R., Shannon R. J., Stewart J. J. P., Tahoces P. G., Vazquez S. A. (2021). AutoMeKin2021:
An open-source program for automated reaction discovery. J. Comput. Chem..

[76] Smith W. R., Missen R. W. (1974). The effect of isomerization on chemical equilibrium. Can. J. Chem. Eng..

[77] Ho, J. ; Coote, M. ; Cramer, C. ; Truhlar, D. Theoretical Calculation of Reduction Potentials. In Organic Electrochemistry; CRC Press, 2015; pp 229–259.

[78] Jensen J. H. (2015). Predicting
accurate absolute binding energies in aqueous solution: thermodynamic
considerations for electronic structure methods. Phys. Chem. Chem. Phys..

[79] Noroozi J., Smith W. R. (2020). Accurately Predicting CO2 Reactive Absorption Properties
in Aqueous Alkanolamine Solutions by Molecular Simulation Requiring
No Solvent Experimental Data. Ind. Eng. Chem.
Res..

[80] Noroozi J., Smith W. R. (2020). Prediction of Alkanolamine pKa Values by Combined Molecular
Dynamics Free Energy Simulations and ab Initio Calculations. J. Chem. Eng. Data.

[81] Ho J., Coote M. L. (2010). A universal approach for continuum solvent pKa calculations:
are we there yet?. Theor. Chem. Acc..

[82] Grimme S. (2012). Supramolecular
Binding Thermodynamics by Dispersion-Corrected Density Functional
Theory. Chem. - Eur. J..

[83] Li Y.-P., Gomes J., Sharada S. M., Bell A. T., Head-Gordon M. (2015). Improved Force-Field
Parameters for QM/MM Simulations of the Energies of Adsorption for
Molecules in Zeolites and a Free Rotor Correction to the Rigid Rotor
Harmonic Oscillator Model for Adsorption Enthalpies. J. Phys. Chem. C.

[84] Scott A. P., Radom L. (1996). Harmonic vibrational frequencies: An evaluation of Hartree-Fock,
Möller-Plesset, quadratic configuration interaction, density
functional theory, and semiempirical scale factors. J. Phys. Chem. A.

[95] Frisch, M. J. ; Trucks, G. W. ; Schlegel, H. B. ; Scuseria, G. E. ; Robb, M. A. ; Cheeseman, J. R. ; Scalmani, G. ; Barone, V. ; Petersson, G. A. ; Nakatsuji, H. ; Li, X. ; Caricato, M. ; Marenich, A. V. ; Bloino, J. ; Janesko, B. G. ; Gomperts, R. ; Mennucci, B. , Hratchian, H. P. ; Ortiz, J. V. ; Izmaylov, A. F. , Sonnenberg, J. L. ; Williams-Young, D. ; Ding, F. ; Lipparini, F. ; Egidi, F. ; Goings, J. ; Peng, B. ; Petrone, A. ; Henderson, T. , Ranasinghe, D. , Zakrzewski, V. G. ; Gao, J. ; Rega, N. ; Zheng, G. ; Liang, W. ; Hada, M. , Ehara, M. ; Toyota, K. ; Fukuda, R. ; Hasegawa, J. ; Ishida, M. ; Nakajima, T. ; Honda, Y. ; Kitao, O. , Nakai, H. ; Vreven, T. ; Throssell, K. ; Montgomery, J. A., Jr. ; Peralta, J. E. ; Ogliaro, F. ; Bearpark, M. J. ; Heyd, J. J. ; Brothers, E. N. ; Kudin, K. N. ; Staroverov, V. N. , Keith, T. A. ; Kobayashi, R. ; Normand, J. ; Raghavachari, K. ; Rendell, A. P. ; Burant, J. C. ; Iyengar, S. S. , Tomasi, J. ; Cossi, M. ; Millam, J. M. ; Klene, M. , Adamo, C. ; Cammi, R. ; Ochterski, J. W. ; Martin, R. L. ; Morokuma, K. ; Farkas, O. ; Foresman, J. B. ; Fox, D. J. Gaussian 16 Revision C.01, (2016), Gaussian Inc.: Wallingford CT.

[96] Barone V., Cimino P., Stendardo E. (2008). Development
and Validation of the
B3LYP/N07D Computational Model for Structural Parameter and Magnetic
Tensors of Large Free Radicals. J. Chem. Theory
Comput..

[85] Scalmani G., Frisch M. J. (2010). Continuous surface charge polarizable continuum models
of solvation. I. General formalism. J. Chem.
Phys..

[86] Wales D. J., Doye J. P. K. (1997). Global Optimization by Basin-Hopping and the Lowest
Energy Structures of Lennard-Jones Clusters Containing up to 110 Atoms. J. Phys. Chem. A.

[87] Goedecker S. (2004). Minima hopping:
An efficient search method for the global minimum of the potential
energy surface of complex molecular systems. J. Chem. Phys..

[97] Pronk S., Pall S., Schulz R., Larsson P., Bjelkmar P., Apostolov R., Shirts M. R., Smith J. C., Kasson P. M., van der Spoel D., Hess B., Lindahl E. (2013). GROMACS 4.5: A high-throughput
and highly parallel open source molecular simulation toolkit. Bioinformatics.

[88] Ebata T., Watanabe T., Mikami N. (1995). Evidence for
the cyclic form of phenol
trimer: Vibrational spectroscopy of the OH stretching vibrations of
jet-cooled phenol dimer and trimer. J. Phys.
Chem. A.

[89] Ohta K., Tominaga K. (2007). Vibrational population relaxation of hydrogen-bonded
phenol complexes in solution: Investigation by ultrafast infrared
pump probe spectroscopy. Chem. Phys..

[90] Fedor A. M., Toda M. (2014). Investigating hydrogen
bonding in phenol using infrared spectroscopy
and computational chemistry. J. Chem. Educ..

[91] Pracht P., Grimme S. (2021). Calculation of absolute
molecular entropies and heat
capacities made simple. Chem. Sci..

[92] Cacelli I., Cimoli A., Livotto P. R., Prampolini G. (2012). An automated
approach for the parameterization of accurate intermolecular force-fields:
Pyridine as a case study. J. Comput. Chem..

[93] Cerezo J., Aranda D., Avila Ferrer F. J., Prampolini G., Santoro F. (2020). Adiabatic-Molecular Dynamics Generalized
Vertical Hessian
Approach: A Mixed Quantum Classical Method To Compute Electronic Spectra
of Flexible Molecules in the Condensed Phase. J. Chem. Theory Comput..

[94] Cerezo J., Santoro F., Prampolini G. (2016). Comparing
classical approaches with
empirical or quantum-mechanically derived force fields for the simulation
electronic lineshapes: application to coumarin dyes. Theor. Chem. Acc..

