# Supplementary material for: Assessing Nonbonded Aggregates Populations: Application to the Concentration-Dependent IR O–H Band of Phenol
Source: J Chem Theory Comput. 2025 Apr 14;21(8):3888–901. doi: 10.1021/acs.jctc.5c00281 (PMC12161665; doi:10.1021/acs.jctc.5c00281)
Supplement: Supplementary file 1 [file ct5c00281_si_001.pdf]

SUPPORTING INFORMATION

Assessing non-bonded aggregates populations: application to the concentration-dependent IR O−H band of phenol

J. Pablo Galvez[a], J. Zúñiga[a] and J. Cerezo[*,b]

[a] *Departamento de Química Física* (Universidad de Murcia),
30100 Murcia, Spain
[b]*Departamento de Química* and
Institute for Advanced Research in Chemical Sciences (IAdChem) (Universidad Autónoma de Madrid),
28049 Madrid, Spain

*javier.cerezo@uam.es

Contents

A Geometric criteria for molecular recognition S3

B Systematic conformational search protocol S8

C Frequencies scaling factor S9

D Dependence of the populations with the thresholds S11

E Convergence of the QM set S13

F QM optimized conformers S16

G Interaction patterns S27

H Equilibrium properties S28

A Geometric criteria for molecular recognition

In Table S1 we present two different sets of thresholds. The values in the set labeled **C/d** correspond to those used to obtain the populations shown throughout this work. The set Loose was used to examine the effects of relaxing the threshold values on the calculation of populations and spectra.

Table S1: Threshold sets used to identify intermolecular interactions between phenol molecules.

	**C/d**	Loose
	Bonds (nm)	
OH···HO	0.26	0.26
OH···Hpi	0.28	0.30
Cpi···Cpi	0.40	0.40
Cpi···Hpi	0.30	0.30
Cpi···HO	0.30	0.30
	Angles (°)	
OH···HO−O	45.0	45.0
OH···Hpi−C	60.0	–
Cpi···Hpi−C	60.0	–
Cpi···HO−O	45.0	45.0

These thresholds were determined by analyzing the pair distribution functions for the corresponding pairs of distance/angle obtained with the TRAVIS [1] program package for post-processing trajectories. Figures S1 to S4 illustrate these functions, computed across the entire trajectory involving 1060 phenol molecules.

Due to the large number of hydrogen-bonded clusters, the pair distribution functions computed from the trajectory primarily reflect their characteristics. Therefore, to derive accurate thresholds for the remaining non-covalent interactions, we computed these distribution functions from the isolated dimer subunits identified in the trajectory using the `aggregate` protocol.

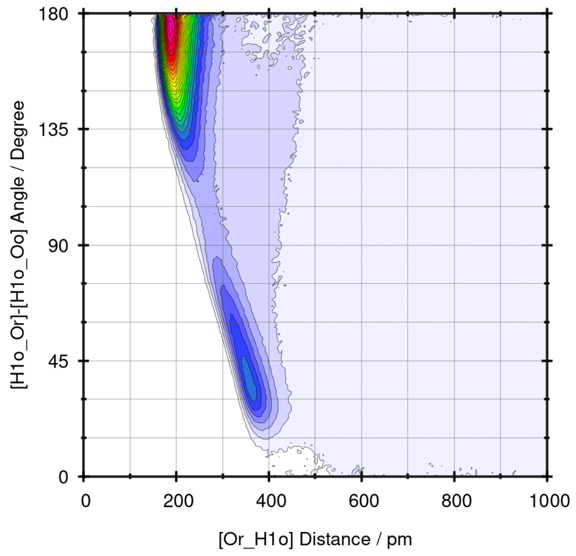

Figure S1: Pair distribution function calculated for the O···HO distance and the O···HO−O angle.

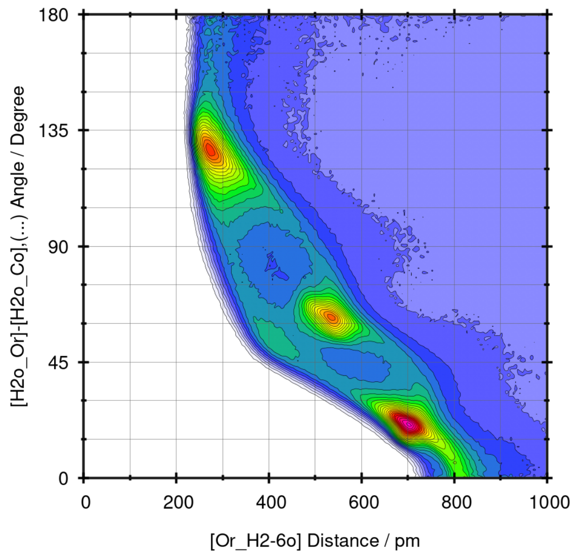

Figure S2: Pair distribution function calculated for the O···Hpi distance and the O···Hpi−C(COM) angle.

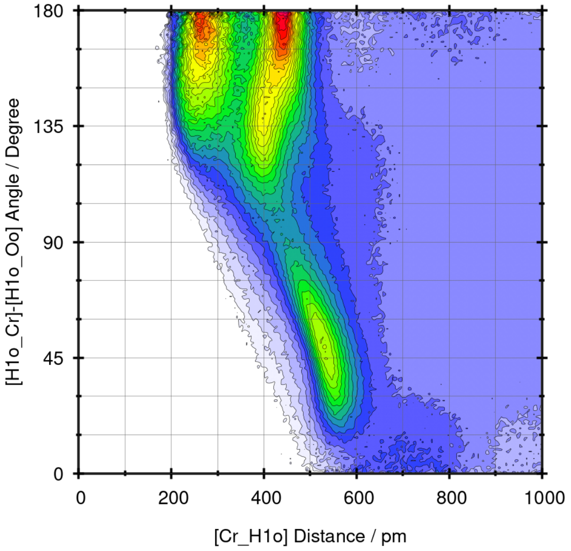

Figure S3: Pair distribution function calculated for the C(COM)┈HO distance and the C(COM)┈HO−O angle.

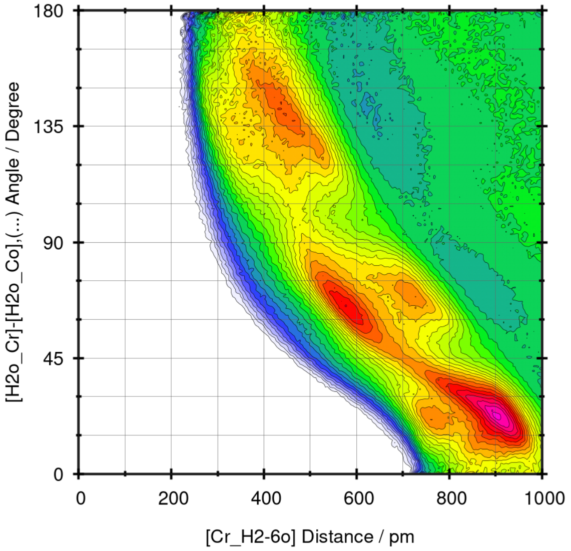

Figure S4: Pair distribution function calculated for the C(COM)┈Hpi distance and the C(COM)┈Hpi−C(COM) angle.

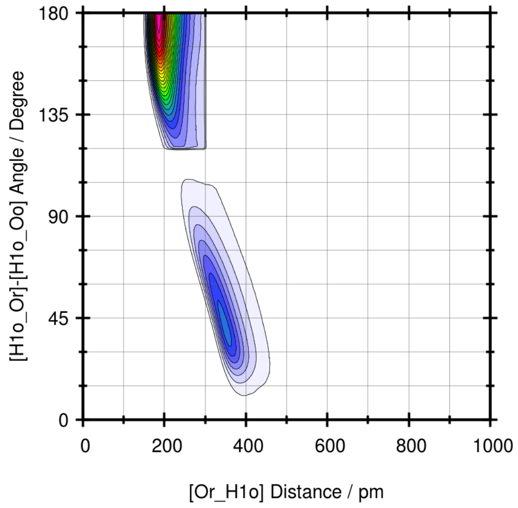

Figure S5: Pair distribution function calculated for the O····HO distance and the O····HO−O angle obtained from the isolated dimer subunits.

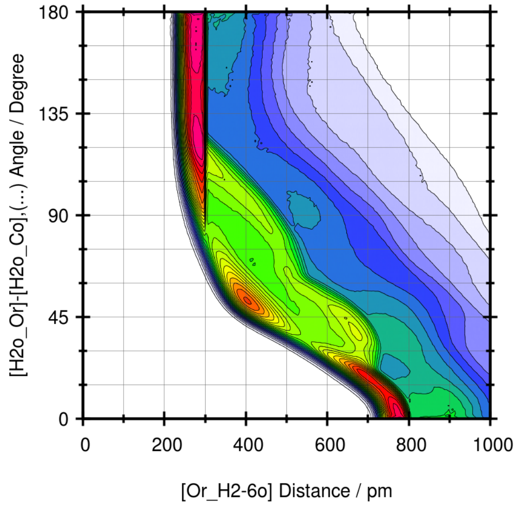

Figure S6: Pair distribution function calculated for the O····Hpi distance and the O····Hpi−C(COM) angle obtained from the isolated dimer subunits.

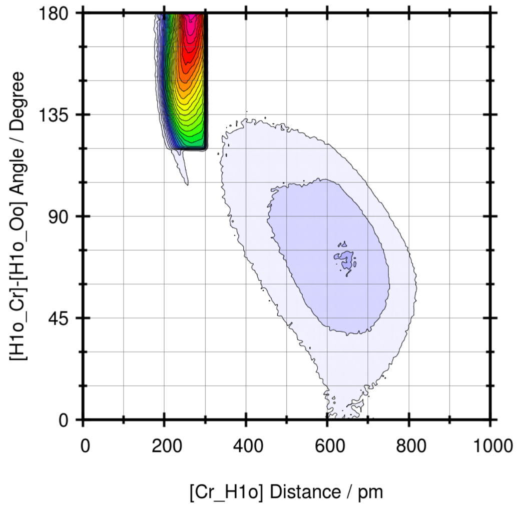

Figure S7: Pair distribution function calculated for the C(COM)···HO distance and the C(COM)···HO−O angle obtained from the isolated dimer subunits.

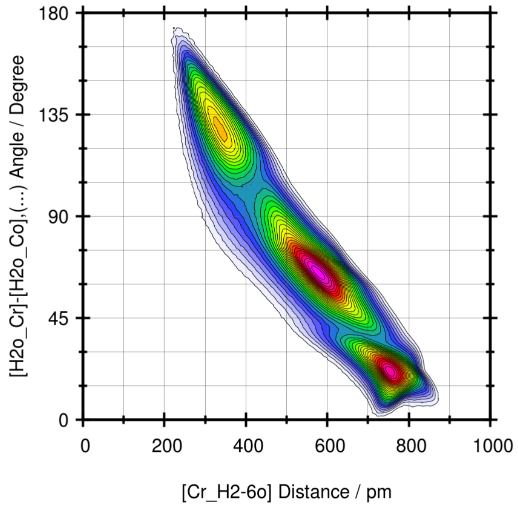

Figure S8: Pair distribution function calculated for the C(COM)···Hpi distance and the C(COM)···Hpi−C(COM) angle obtained from the isolated dimer subunits.

B Systematic conformational search protocol

The procedure followed in this work to obtain the set of candidate structures from an automated conformational search involved the following stages;

1. We generate a first set of candidate structures with the `aggregation` module of *AutoMeKin*. By combining monomer(s) and/or high-order aggregates, we generate the initial candidate structures: 1000 dimers, 1400 trimers and 1400 tetramers.

2. The *AutoMeKin* protocol incorporates a pre-optimization step carried out using a semi-empirical level of theory. The modification to this protocol adopted in the current study involves replacing this step with a partial optimization conducted directly at the high level of theory prior to the screening and full optimization of the structures. Consequently, geometries optimizations were performed on the candidate structures at the PCM/B3LYP-D3/N07D level of theory using the default grid and convergence criteria of *Gaussian16*, limiting the number of optimization steps to 100.

3. This first set of partially optimized geometries was screened to to remove repeated and redundant structures (related by permutations of phenol fragments), using a Fortran code developed locally.

4. From the previous set, the Boltzmann factor $e^{-(E_i - E_0)/KT}$ for each geometry was calculated, where E_0 is the energy of the lowest energy conformer. Those structures with a Boltzmann factor greater than 0.01 (44 dimers, 313 trimers and 127 tetramers) were further subjected to geometry optimization with tighter grid and convergence criteria (keywords `opt=tight` and `grid=ultrafine`).

5. After a second screening to remove duplicate conformations and/or rotamers, only those structures with a Boltzmann factor greater than 0.01 (44 dimers, 136 trimers and 91 tetramers) were taken for the subsequent frequency calculation. The calculation of their vibrational frequencies allowed the confirmation of each structure as a true minimum on their respective PES.

6. Finally, only those structures with a population $e^{-(G_i-G_0)/KT}$ greater than 0.01 (38 dimers, 133 trimers, 75 tetramers), with G_0 being the free energy of the lowest energy conformer, where further considered for the calculation of the equilibrium constants and harmonic IR spectra.

C Frequencies scaling factor

In this work, we derived frequencies scaling factores from the experimental frequencies of phenol monomer in gas-phase and in carbon tetraclhoride, and the harmonic frequencies obtained at the B3LYP-D3/N07D level of theory. The values used for the fundamental frequencies are listed in Table S2.

Frequency scaling factors were determined using Equation (1) [2] and the aforementioned fundamental frequencies,

$$\lambda = \frac{\sum_i^{N_{freq}} \nu_i \omega_i}{\sum_i^{N_{freq}} \omega_i^2} \tag{1}$$

In this equation, N_{freq} represents the number of vibrational frequencies considered in the fitting process, ν denotes the fundamental frequency measured experimentally, and ω corresponds to the harmonic frequency calculated in this work.

Table S2: Fundamental frequencies of phenol monomer in gas-phase and CCl_4 solution.

Gas-phase[a]		CCl_4 solution[b]	
Harmonic	Experimental	Harmonic	Experimental
3833.917	3656	3823.48	3611
3212.76	3087	3211.56	3092
3206.424	3070	3205.12	3076
3193.015	3063	3193.06	3044
3184.429	3049	3184.55	3019
3166.335	3027	–	–
1651.556	1610	1648.38	1605
1640.491	1604	1637.67	1598
1529.499	1505	1526.42	1499
1501.709	1472	1499.78	1470
1369.899	1343	1367.75	1342
1358.105	1277	1356.28	1330
1282.247	1261	1277.06	1257
1197.296	1174	1193.04	1179
1191.659	1169	1190.68	1167
1183.536	1150	1182.08	1151
1096.637	1070	1093.84	1069
1043.92	1026	1042.21	1024
1009.268	999	1007.81	1000
985.951	995	–	–
966.893	973	987.059	977
892.423	881	892.148	883
827.751	820	826.086	826
826.004	817	825.549	811
762.669	751	762.509	750
695.965	686	694.622	689
629.551	618	628.59	620
533.919	526	533.011	529
514.352	504	513.822	506
420.738	409	419.611	420
410.909	404	404.858	405
329.12	309	323.193	322
229.205	225	225.488	235

[a] From Ref. [3]
[b] From Ref. [4]

D Dependence of the populations with the thresholds

In Table S3, we compare the **C/d** populations obtained using the two threshold sets described in Table S1. Additionally, Figure S9 presents a comparison of the calculated IR spectra derived from these two sets of populations, and the equilibrium constants from Table S6.

Table S3: Populations of the different conformers computed using the classical MD methodology with two different threshold sets.

	Dimer[a]	
	**C/d**	Loose
OH-bonded	68.65	57.84
$\pi - \pi$	8.58	8.00
OH - π	22.76	34.16
	Trimer[b]	
	**C/d**	Loose
Dimer-like	41.42	46.56
Linear	48.67	36.70
Cyclic	1.40	1.15
$\pi - \pi$ bonded	4.13	5.57
OH-π bonded	4.41	10.03
	Tetramer[b]	
	**C/d**	Loose
Dimer-like	15.41	23.62
Double dimer	10.59	10.27
Trimer-like linear	26.80	27.51
Trimer-like cycle	0.47	0.54
Star	0.38	0.26
Linear	34.53	25.12
Cocktail	0.09	0.07
Cyclic	9.68	7.50
$\pi - \pi$ bonded	1.30	2.54
OH-π bonded	0.75	2.59

[a] Computed averaging the populations of dimer conformations of every MD simulation.
[b] Computed averaging the populations of the aggregate conformations of the two MD simulations at higher concentration.

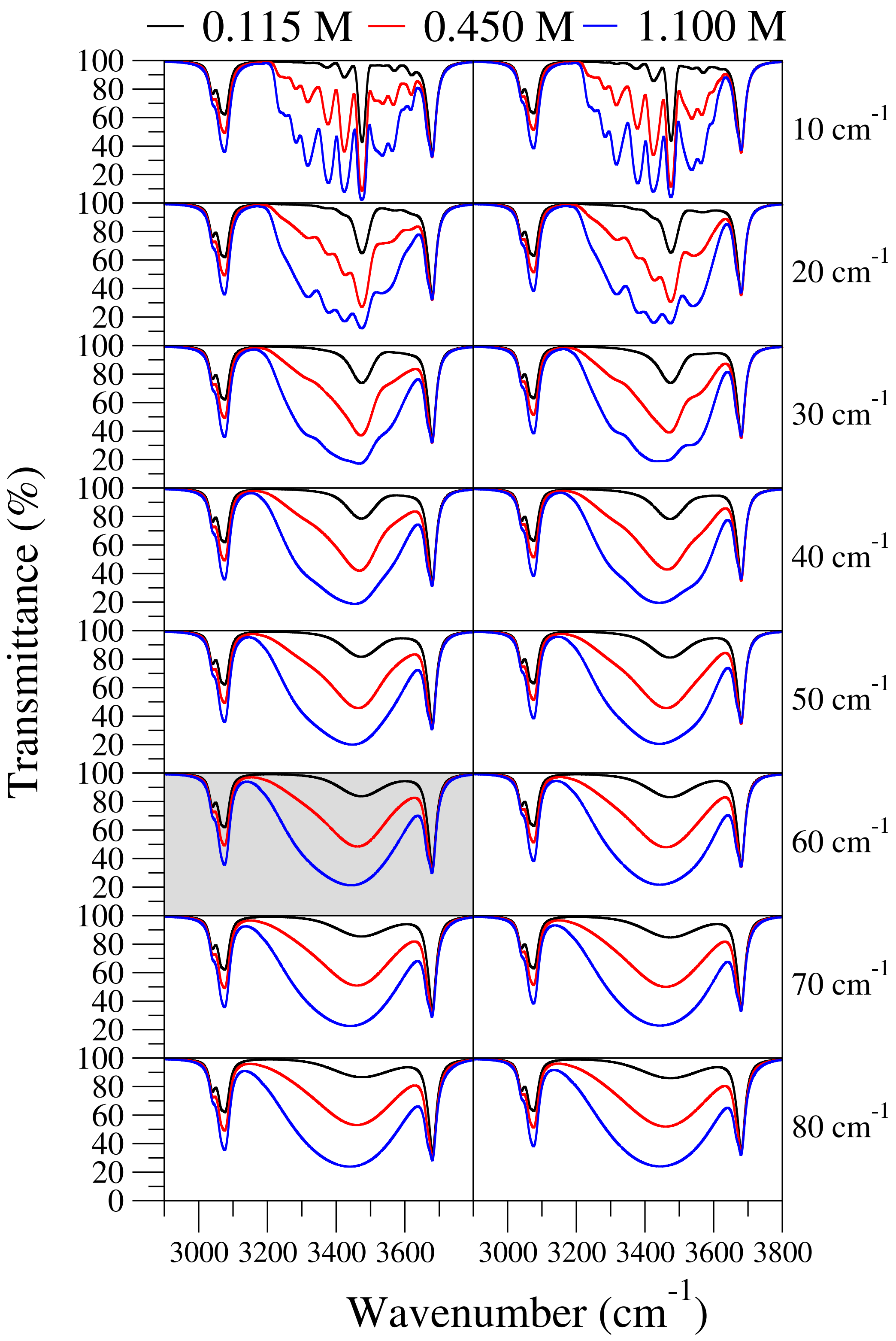

Figure S9: IR spectra calculated using the **C/d** methodology for two different threshold sets and several HWHM values. First column includes spectra computed with the thresholds used in the main text and second column includes those with the *Loose* thresholds (see Table S1). The spectrum highlighted in gray corresponds to the C/d simulation shown in Fig. 5 in the main text.

E Convergence of the QM set

In this section, we present the results of the **Q/s** methodology applied to two distinct conformer sets. Table S4 provides the populations of the conformers calculated using both sets, where the first set is smaller due to insufficient conformational sampling, and the second set includes the conformers from the first set along with an additional subset of conformers optimized from MD trajectories. Figure S10 shows the average IR spectra computed for these conformer sets across various HWHM values, highlighting the influence of the conformer set size and spectral broadening on the final spectral profiles. Additionally, Figure S11 illustrates the impact of conformer set size and HWHM on the resulting spectra obtained with the **C/d** protocol.

Table S4: Populations of the different conformers computed using the **Q/s** methodology with two different conformer sets.

	Dimer	
	**Q/s**	Small
OH-bonded	51.00	51.00
$\pi - \pi$	33.43	33.43
OH - π	15.56	15.56
	Trimer	
	**Q/s**	Small
Dimer-like	55.78	57.09
Linear	23.82	24.57
Cyclic	17.79	18.34
$\pi - \pi$ bonded	2.55	-
OH-π bonded	-	-
	Tetramer	
	**Q/s**	Small
Dimer-like	0.18	-
Double dimer	3.67	3.84
Trimer-like linear	5.24	0.89
Trimer-like cycle	0.11	0.10
Star	0.04	0.04
Linear	32.32	37.53
Cocktail	3.51	0.01
Cyclic	54.96	57.61
$\pi - \pi$ bonded	0.01	-
OH-π bonded	-	-

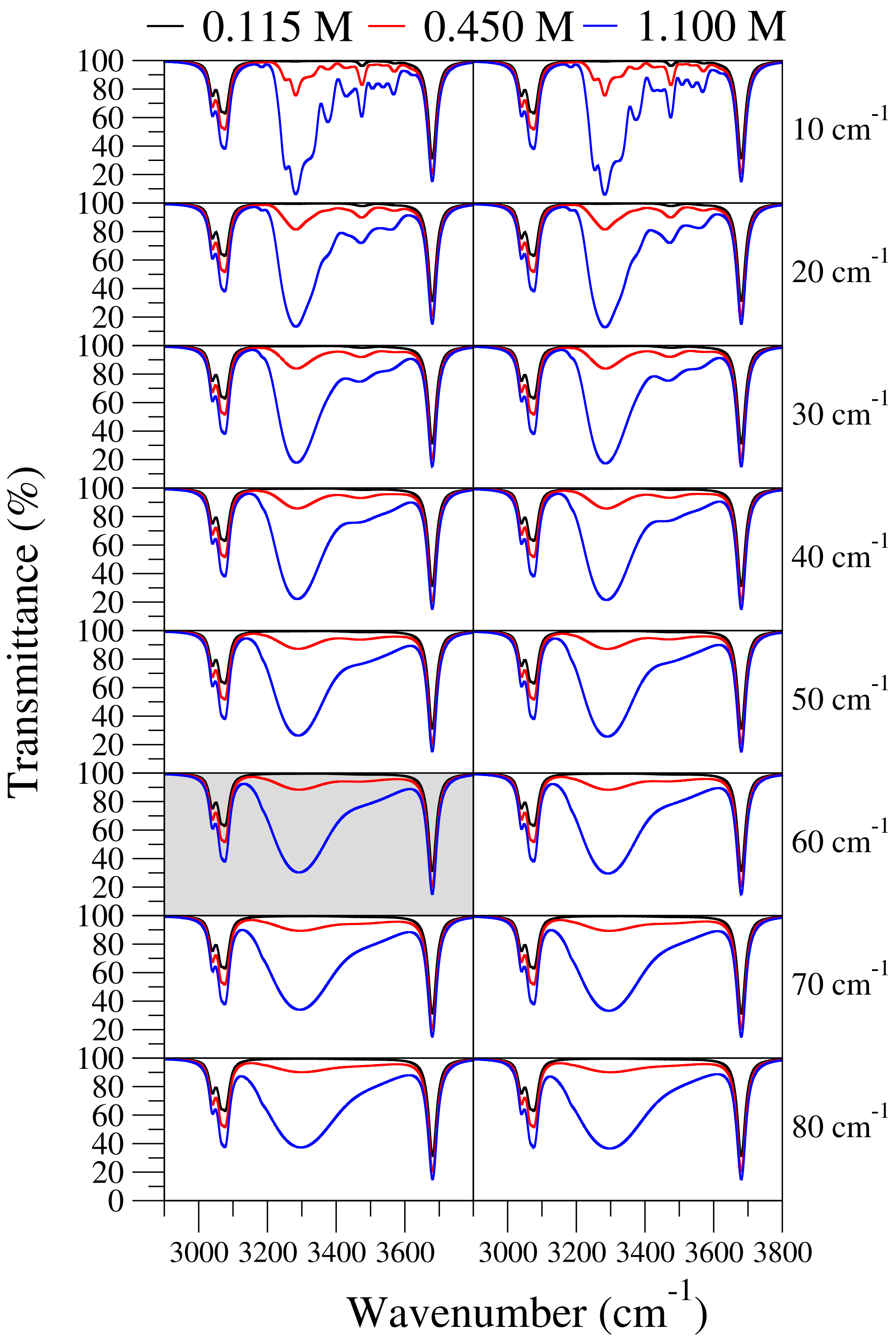

Figure S10: Average IR spectra computed using the **Q/s** methodology for two conformer sets and several HWHM values.

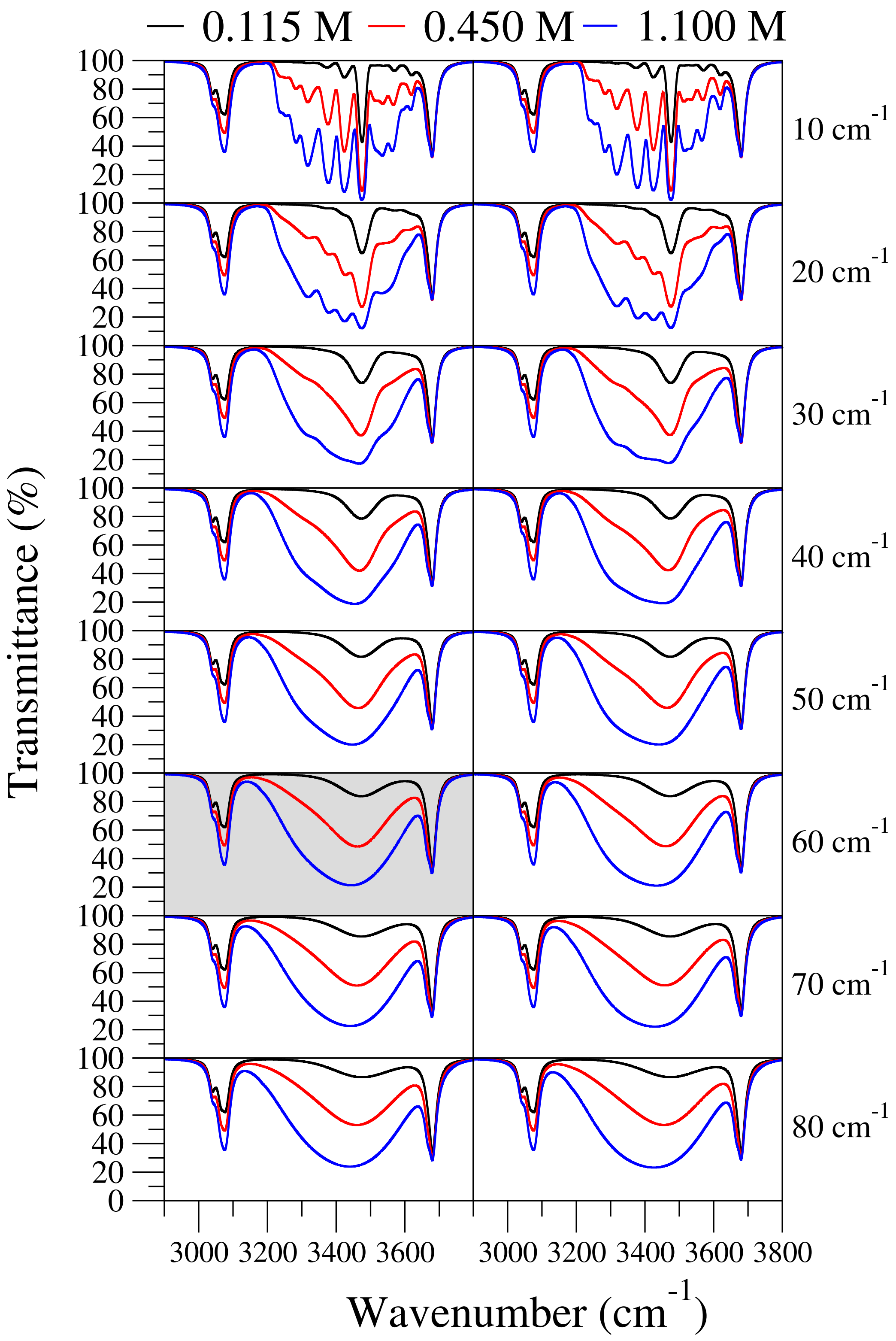

Figure S11: Average IR spectra computed using the **C/d** methodology for two conformer sets and several HWHM values.

F QM optimized conformers

We here summarize the contributions to the free energy for the QM-optimized conformers, alongside the interaction patterns assigned to each configuration. These patterns provide a detailed characterization of the conformers, linking their structural and spectroscopic properties. Additionally, Figures S12 to S14 illustrate the IR spectra for each conformer within the interaction patterns, as well as the average IR spectrum corresponding to each pattern. This comparison highlights the spectral small variations among conformers and their collective representation within each interaction pattern.

	Monomer				
Name	$E_{gas}^{\text{N07Tdiff}}$(CP)	$E_{gas}^{\text{N07Tdiff}}$	$E_{CCl_4}^{\text{N07Tdiff}}$	$\delta G_{i,VRT}^{*,\text{N07D}}$	Pattern
monomer	-	-	-307.601144843	0.07119820	-
	Dimer				
Name	$E_{gas}^{\text{N07Tdiff}}$(CP)	$E_{gas}^{\text{N07Tdiff}}$	$E_{CCl_4}^{\text{N07Tdiff}}$	$\delta G_{i,VRT}^{*,\text{N07D}}$	Pattern
dimer 1	-615.20868552	-615.20895415	-615.21351695	0.16014611	OH-bonded
dimer 2	-615.20863868	-615.20890867	-615.21332592	0.16029674	OH-bonded
dimer 3	-615.20697516	-615.20728559	-615.21176307	0.15933762	π-π bonded
dimer 4	-615.20659691	-615.20689549	-615.21133610	0.15901044	π-π bonded
dimer 5	-615.20638100	-615.20669617	-615.21123736	0.15923000	π-π bonded
dimer 6	-615.20374934	-615.20399546	-615.20861020	0.15861290	π-π bonded
dimer 7	-615.20302950	-615.20326155	-615.20804371	0.15852850	π-π bonded
dimer 8	-615.20187670	-615.20209240	-615.20700563	0.15758529	π-π bonded
dimer 9	-615.20291445	-615.20314121	-615.20791624	0.15856861	π-π bonded
dimer 10	-615.20235257	-615.20258397	-615.20745271	0.15809849	π-π bonded
dimer 11	-615.20230897	-615.20254149	-615.20742648	0.15807748	π-π bonded
dimer 12	-615.20291639	-615.20314568	-615.20793699	0.15863752	π-π bonded
dimer 13	-615.20308634	-615.20332009	-615.20809596	0.15888053	π-π bonded
dimer 14	-615.20207634	-615.20231421	-615.20721145	0.15799452	π-π bonded
dimer 15	-615.20300302	-615.20325180	-615.20805517	0.15894112	π-π bonded
dimer 16	-615.20311649	-615.20335497	-615.20812169	0.15912009	π-π bonded
dimer 17	-615.20235690	-615.20266308	-615.20733387	0.15831422	π-π bonded
dimer 18	-615.20154954	-615.20176399	-615.20678703	0.15786388	π-π bonded
dimer 19	-615.20155625	-615.20177092	-615.20678879	0.15794929	π-π bonded
dimer 20	-615.20233100	-615.20256627	-615.20746214	0.15878328	π-π bonded
dimer 21	-615.20176226	-615.20198078	-615.20696361	0.15831888	π-π bonded
dimer 22	-615.20184525	-615.20213046	-615.20683935	0.15825752	π-π bonded

Name	$E_{gas}^{\mathrm{N07Tdiff}}$(CP)	$E_{gas}^{\mathrm{N07Tdiff}}$	$E_{CCl_4}^{\mathrm{N07Tdiff}}$	$\delta G_{i,VRT}^{*,\mathrm{N07D}}$	Pattern
dimer 23	-615.20675577	-615.20705347	-615.21133735	0.15898863	OH-π bonded
dimer 24	-615.20721771	-615.20746307	-615.21081412	0.15972015	OH-π bonded
dimer 25	-615.20682184	-615.20710294	-615.21050418	0.16022304	OH-π bonded
dimer 26	-615.20223681	-615.20242265	-615.20689600	0.15768846	OH-π bonded
dimer 27	-615.20204465	-615.20222638	-615.20669773	0.15815297	OH-π bonded
dimer 28	-615.20208531	-615.20227620	-615.20669060	0.15834305	OH-π bonded
dimer 29	-615.20148992	-615.20165648	-615.20617977	0.15791723	OH-π bonded
dimer 30	-615.20143836	-615.20160431	-615.20613546	0.15802551	OH-π bonded
dimer 31	-615.20137911	-615.20156290	-615.20623749	0.15811870	OH-π bonded
dimer 32	-615.20153688	-615.20171070	-615.20621689	0.15816669	OH-π bonded
dimer 33	-615.20153505	-615.20170029	-615.20623313	0.15831083	OH-π bonded
dimer 34	-615.20149035	-615.20168164	-615.20622485	0.15831327	OH-π bonded
dimer 35	-615.20149634	-615.20168245	-615.20627726	0.15841295	OH-π bonded
dimer 36	-615.20146438	-615.20162812	-615.20616895	0.15834191	OH-π bonded
dimer 37	-615.20177113	-615.20199081	-615.20661777	0.15924539	OH-π bonded
dimer 38	-615.20138089	-615.20157754	-615.20605578	0.15827570	OH-π bonded

	Trimer				
Name	$E_{gas}^{\mathrm{N07Tdiff}}$(CP)	$E_{gas}^{\mathrm{N07Tdiff}}$	$E_{CCl_4}^{\mathrm{N07Tdiff}}$	$\delta G_{i,VRT}^{*,\mathrm{N07D}}$	Pattern
trimer 1	-922.82862813	-922.82947922	-922.83321859	0.25222407	Dimer-like
trimer 2	-922.82846641	-922.82932469	-922.83303487	0.25233179	Dimer-like
trimer 3	-922.82610383	-922.82686477	-922.83072274	0.25107828	Dimer-like
trimer 4	-922.82696305	-922.82783319	-922.83165364	0.25231571	Dimer-like
trimer 5	-922.82735169	-922.82820475	-922.83193778	0.25264467	Dimer-like
trimer 6	-922.82663519	-922.82744938	-922.83150955	0.25237233	Dimer-like
trimer 7	-922.82612932	-922.82693222	-922.83106124	0.25199438	Dimer-like
trimer 8	-922.82557208	-922.82632834	-922.83036204	0.25143671	Dimer-like
trimer 9	-922.82598364	-922.82680594	-922.83105589	0.25226195	Dimer-like
trimer 10	-922.82487276	-922.82563779	-922.82985728	0.25116514	Dimer-like
trimer 11	-922.82404454	-922.82482895	-922.83006421	0.25187149	Dimer-like
trimer 12	-922.82184712	-922.82263221	-922.82773759	0.24974790	Dimer-like
trimer 13	-922.82336040	-922.82414704	-922.82935585	0.25146954	Dimer-like
trimer 14	-922.82266526	-922.82337049	-922.82818075	0.25051416	Dimer-like
trimer 15	-922.82405512	-922.82484727	-922.82960934	0.25194027	Dimer-like
trimer 16	-922.82342750	-922.82422104	-922.82912706	0.25150675	Dimer-like
trimer 17	-922.82363085	-922.82441418	-922.82916108	0.25155520	Dimer-like
trimer 18	-922.82328898	-922.82407116	-922.82885835	0.25129596	Dimer-like
trimer 19	-922.82403184	-922.82483657	-922.82998821	0.25241232	Dimer-like
trimer 20	-922.82369981	-922.82448309	-922.82923741	0.25171211	Dimer-like
trimer 21	-922.82276205	-922.82353067	-922.82868632	0.25128234	Dimer-like

Name	$E_{gas}^{\mathrm{N07Tdiff}}$(CP)	$E_{gas}^{\mathrm{N07Tdiff}}$	$E_{CCl_4}^{\mathrm{N07Tdiff}}$	$\delta G_{i,VRT}^{*,\mathrm{N07D}}$	Pattern
trimer 22	-922.82307380	-922.82387151	-922.82912011	0.25169507	Dimer-like
trimer 23[a]	-922.82062650	-922.82132802	-922.82665992	0.24940357	Dimer-like
trimer 24	-922.82319543	-922.82398715	-922.82880723	0.25160523	Dimer-like
trimer 25	-922.82189219	-922.82260331	-922.82758314	0.25047814	Dimer-like
trimer 26	-922.82165622	-922.82241795	-922.82770891	0.25055689	Dimer-like
trimer 27	-922.82323651	-922.82404148	-922.82934976	0.25218945	Dimer-like
trimer 28	-922.82330845	-922.82411430	-922.82907553	0.25210051	Dimer-like
trimer 29	-922.82172191	-922.82253230	-922.82767327	0.25079634	Dimer-like
trimer 30	-922.82274957	-922.82353954	-922.82831459	0.25159979	Dimer-like
trimer 31	-922.82057115	-922.82125391	-922.82647493	0.24987180	Dimer-like
trimer 32	-922.82130192	-922.82197348	-922.82687022	0.25050177	Dimer-like
trimer 33	-922.82038708	-922.82116078	-922.82769672	0.25124631	Dimer-like
trimer 34	-922.82022528	-922.82095422	-922.82703507	0.25058366	Dimer-like
trimer 35	-922.82173325	-922.82244521	-922.82731505	0.25088992	Dimer-like
trimer 36	-922.82118980	-922.82196624	-922.82820750	0.25181088	Dimer-like
trimer 37	-922.82075020	-922.82142670	-922.82636861	0.25030320	Dimer-like
trimer 38	-922.82113238	-922.82179076	-922.82634977	0.25054127	Dimer-like
trimer 39	-922.82040959	-922.82113798	-922.82721690	0.25141257	Dimer-like
trimer 40	-922.82028741	-922.82098569	-922.82618115	0.25049632	Dimer-like
trimer 41	-922.81954816	-922.82032929	-922.82661184	0.25091607	Dimer-like
trimer 42	-922.81976358	-922.82049784	-922.82657864	0.25086250	Dimer-like
trimer 43	-922.82010683	-922.82085511	-922.82608437	0.25040330	Dimer-like
trimer 44	-922.82066603	-922.82137684	-922.82651320	0.25087567	Dimer-like
trimer 45	-922.81895917	-922.81963339	-922.82574116	0.25037536	Dimer-like
trimer 46	-922.81935845	-922.82001263	-922.82524363	0.24980830	Dimer-like
trimer 47	-922.81961112	-922.82032652	-922.82603710	0.25052900	Dimer-like
trimer 48	-922.81980847	-922.82053508	-922.82621720	0.25077577	Dimer-like
trimer 49	-922.82060835	-922.82140741	-922.82655897	0.25113679	Dimer-like
trimer 50	-922.81968908	-922.82048969	-922.82669481	0.25134762	Dimer-like
trimer 51	-922.82094419	-922.82175055	-922.82658564	0.25117093	Dimer-like
trimer 52[a]	-922.81901201	-922.81972910	-922.82593024	0.25058630	Dimer-like
trimer 53	-922.81963077	-922.82035155	-922.82609997	0.25081397	Dimer-like
trimer 54	-922.81912921	-922.81984303	-922.82641434	0.25117044	Dimer-like
trimer 55	-922.81950986	-922.82021791	-922.82600800	0.25081847	Dimer-like
trimer 56	-922.81872660	-922.81941424	-922.82526730	0.25041888	Dimer-like
trimer 57	-922.81948221	-922.82014653	-922.82549925	0.25073138	Dimer-like
trimer 58	-922.81838672	-922.81915855	-922.82536237	0.25079976	Dimer-like
trimer 59[a]	-922.82012821	-922.82085367	-922.82576098	0.25098486	Dimer-like
trimer 60	-922.81959239	-922.82030344	-922.82592508	0.25114773	Dimer-like
trimer 61	-922.81862355	-922.81931930	-922.82527711	0.25052602	Dimer-like
trimer 62	-922.81939929	-922.82010681	-922.82599242	0.25126603	Dimer-like

Name	$E_{gas}^{\text{N07Tdiff}}$(CP)	$E_{gas}^{\text{N07Tdiff}}$	$E_{CCl_4}^{\text{N07Tdiff}}$	$\delta G_{i,VRT}^{*,\text{N07D}}$	Pattern
trimer 63	-922.81828080	-922.81902344	-922.82523853	0.25069987	Dimer-like
trimer 64	-922.81959993	-922.82032377	-922.82623881	0.25171666	Dimer-like
trimer 65	-922.81845110	-922.81914452	-922.82516793	0.25061039	Dimer-like
trimer 66	-922.81815076	-922.81885496	-922.82489607	0.25032758	Dimer-like
trimer 67	-922.81854451	-922.81926615	-922.82530461	0.25087054	Dimer-like
trimer 68	-922.81867706	-922.81939161	-922.82541475	0.25093460	Dimer-like
trimer 69	-922.81730062	-922.81800073	-922.82463868	0.25027368	Dimer-like
trimer 70	-922.81827223	-922.81899552	-922.82513088	0.25070718	Dimer-like
trimer 71	-922.81875339	-922.81947659	-922.82535963	0.25097580	Dimer-like
trimer 72	-922.81804821	-922.81874797	-922.82494644	0.25060461	Dimer-like
trimer 73	-922.82012465	-922.82086649	-922.82567390	0.25102699	Dimer-like
trimer 74	-922.81569645	-922.81629812	-922.82280668	0.24909924	Dimer-like
trimer 75	-922.81634317	-922.81702289	-922.82375189	0.25019857	Dimer-like
trimer 76	-922.81685919	-922.81752833	-922.82358043	0.25030048	Dimer-like
trimer 77	-922.81826473	-922.81902736	-922.82523867	0.25095952	Dimer-like
trimer 78	-922.81623456	-922.81687341	-922.82351647	0.24971575	Dimer-like
trimer 79	-922.81607385	-922.81676344	-922.82389442	0.25011886	Dimer-like
trimer 80	-922.81622491	-922.81691893	-922.82396641	0.25026755	Dimer-like
trimer 81	-922.81814241	-922.81893941	-922.82532008	0.25149415	Dimer-like
trimer 82	-922.81607598	-922.81676208	-922.82378674	0.25019368	Dimer-like
trimer 83	-922.81811954	-922.81886479	-922.82510624	0.25171371	Dimer-like
trimer 84	-922.81832451	-922.81909373	-922.82537804	0.25176744	Dimer-like
trimer 85	-922.81626219	-922.81695559	-922.82398775	0.25057677	Dimer-like
trimer 86	-922.81608590	-922.81675987	-922.82382989	0.25052738	Dimer-like
trimer 87	-922.81642863	-922.81714698	-922.82398241	0.24974791	Dimer-like
trimer 88	-922.81827610	-922.81898617	-922.82510150	0.25082657	Dimer-like
trimer 89	-922.81829620	-922.81901219	-922.82513398	0.25109213	Dimer-like
trimer 90	-922.81785566	-922.81854245	-922.82468607	0.25053439	Dimer-like
trimer 91	-922.81800654	-922.81870220	-922.82472964	0.25079207	Dimer-like
trimer 92	-922.81787752	-922.81858068	-922.82477372	0.25073301	Dimer-like
trimer 93	-922.81665881	-922.81735520	-922.82425426	0.25033834	Dimer-like
trimer 94	-922.81692855	-922.81762969	-922.82449190	0.25068481	Dimer-like
trimer 95	-922.81807627	-922.81873909	-922.82473952	0.25105882	Dimer-like
trimer 96	-922.81771732	-922.81840542	-922.82453499	0.25074581	Dimer-like
trimer 97[a]	-922.81853233	-922.81926949	-922.82529024	0.25143126	Dimer-like
trimer 98[a]	-922.81628865	-922.81697934	-922.82391508	0.25041313	Dimer-like
trimer 99	-922.81805579	-922.81878769	-922.82477769	0.25165151	Dimer-like
trimer 100	-922.81533440	-922.81596416	-922.82241447	0.24932748	Dimer-like
trimer 101	-922.81352985	-922.81403804	-922.82067000	0.24837613	Dimer-like
trimer 102	-922.81475147	-922.81539177	-922.82191004	0.24969247	Dimer-like
trimer 103	-922.81367862	-922.81419045	-922.82084272	0.24855923	Dimer-like

Name	$E_{gas}^{\mathrm{N07Tdiff}}$(CP)	$E_{gas}^{\mathrm{N07Tdiff}}$	$E_{CCl_4}^{\mathrm{N07Tdiff}}$	$\delta G_{i,VRT}^{*,\mathrm{N07D}}$	Pattern
trimer 104	-922.81355378	-922.81406015	-922.82070917	0.24859217	Dimer-like
trimer 105	-922.81197930	-922.81246807	-922.81940984	0.24754500	Dimer-like
trimer 106	-922.81479723	-922.81546031	-922.82200404	0.25022154	Dimer-like
trimer 107	-922.81299844	-922.81350137	-922.82030146	0.24869503	Dimer-like
trimer 108	-922.81456581	-922.81521682	-922.82178511	0.25063674	Dimer-like
trimer 109	-922.81437890	-922.81504550	-922.82151429	0.25037149	Dimer-like
trimer 110[a]	-922.81278149	-922.81330414	-922.82023572	0.24857239	Dimer-like
trimer 111[a]	-922.81281886	-922.81334815	-922.82023735	0.24900838	Dimer-like
trimer 112	-922.81599961	-922.81668902	-922.82370256	0.25050158	Dimer-like
trimer 113	-922.81710261	-922.81783897	-922.82401106	0.25118073	Dimer-like
trimer 114	-922.81602715	-922.81672927	-922.82357666	0.25094717	Dimer-like
trimer 115	-922.81608550	-922.81683240	-922.82329533	0.25050519	Dimer-like
trimer 116	-922.81637570	-922.81702034	-922.82350300	0.25082522	Dimer-like
trimer 117	-922.81617314	-922.81691483	-922.82345560	0.25084424	Dimer-like
trimer 118	-922.81650098	-922.81719242	-922.82408430	0.25176896	Dimer-like
trimer 119	-922.81489142	-922.81552054	-922.82241602	0.25001094	Dimer-like
trimer 120	-922.81771239	-922.81843358	-922.82398268	0.25094228	Dimer-like
trimer 121	-922.81644364	-922.81711469	-922.82331908	0.25050023	Dimer-like
trimer 122	-922.81550825	-922.81618156	-922.82223180	0.25044955	Dimer-like
trimer 123[a]	-922.81145410	-922.81189659	-922.81855761	0.24827661	Dimer-like
trimer 124	-922.82902250	-922.82977021	-922.83335712	0.25317205	Linear
trimer 125	-922.82814019	-922.82889728	-922.83257805	0.25262931	Linear
trimer 126	-922.82768866	-922.82841843	-922.83215019	0.25282184	Linear
trimer 127	-922.82542060	-922.82621260	-922.83127483	0.25253600	Linear
trimer 128	-922.82388803	-922.82459863	-922.83059035	0.25197030	Linear
trimer 129	-922.82546667	-922.82624265	-922.83147859	0.25294878	Linear
trimer 130	-922.82251669	-922.82318871	-922.82868564	0.25027175	Linear
trimer 131	-922.82577280	-922.82654420	-922.83164380	0.25321147	Linear
trimer 132	-922.82374617	-922.82451421	-922.82993914	0.25237101	Linear
trimer 133	-922.82130190	-922.82191591	-922.82774926	0.25058626	Linear
trimer 134	-922.82247110	-922.82312560	-922.82860300	0.25158177	Linear
trimer 135	-922.82210224	-922.82273841	-922.82819767	0.25149419	Linear
trimer 136	-922.82133200	-922.82195422	-922.82776683	0.25138282	Linear
trimer 137	-922.81926043	-922.81995948	-922.82623204	0.25157148	Linear
trimer 138	-922.81600821	-922.81661253	-922.82377462	0.24979629	Linear
trimer 139	-922.82012519	-922.82090662	-922.82570762	0.25241989	Linear
trimer 140	-922.83090722	-922.83170901	-922.83487571	0.25407633	Cyclic
trimer 141	-922.82746200	-922.82811230	-922.83142041	0.25176446	Cyclic
trimer 142[a]	-922.82542602	-922.82629905	-922.83041505	0.25147877	π-π bonded
trimer 143[a]	-922.82311591	-922.82394660	-922.82852735	0.25163180	π-π bonded
trimer 144[a]	-922.82184635	-922.82266853	-922.82767742	0.25137326	π-π bonded

Name	$E_{gas}^{\mathrm{N07Tdiff}}$(CP)	$E_{gas}^{\mathrm{N07Tdiff}}$	$E_{CCl_4}^{\mathrm{N07Tdiff}}$	$\delta G_{i,VRT}^{*,\mathrm{N07D}}$	Pattern
trimer 145[a]	-922.82136722	-922.82217754	-922.82715636	0.25122736	π-π bonded
trimer 146[a]	-922.82134438	-922.82215261	-922.82714610	0.25117126	π-π bonded
trimer 147[a]	-922.82173558	-922.82258656	-922.82753117	0.25160906	π-π bonded
trimer 148[a]	-922.82109971	-922.82194146	-922.82708559	0.25133170	π-π bonded
trimer 149[a]	-922.82138199	-922.82220668	-922.82718143	0.25144540	π-π bonded
trimer 150[a]	-922.82063814	-922.82147029	-922.82649646	0.25068947	π-π bonded
trimer 151[a]	-922.82087492	-922.82167235	-922.82664671	0.25104449	π-π bonded
trimer 152[a]	-922.82013000	-922.82092388	-922.82606256	0.25108130	π-π bonded
trimer 153[a]	-922.81999574	-922.82084174	-922.82602934	0.25151916	π-π bonded
trimer 154[a]	-922.81408943	-922.81468790	-922.82123399	0.24798745	π-π bonded
trimer 155[a]	-922.81362127	-922.81424698	-922.82092680	0.24765626	π-π bonded
trimer 156[a]	-922.81746972	-922.81821717	-922.82386654	0.25039694	π-π bonded
trimer 157[a]	-922.81357306	-922.81423983	-922.82058442	0.24872400	π-π bonded
trimer 158[a]	-922.81402469	-922.81463794	-922.82079860	0.24800523	π-π bonded
trimer 159[a]	-922.81108120	-922.81161003	-922.81844546	0.24777553	π-π bonded
trimer 160[a]	-922.81363412	-922.81430665	-922.82079902	0.24947648	π-π bonded
trimer 161[a]	-922.81365396	-922.81432225	-922.82085888	0.24968065	π-π bonded
trimer 162[a]	-922.81044319	-922.81098234	-922.81789458	0.24742974	π-π bonded
trimer 163[a]	-922.81390784	-922.81457852	-922.82098070	0.24915072	π-π bonded
trimer 164[a]	-922.80943950	-922.81006658	-922.81693482	0.24908730	π-π bonded
trimer 165[a]	-922.81025178	-922.81084354	-922.81746757	0.24841482	π-π bonded
trimer 166[a]	-922.81700724	-922.81774072	-922.82349912	0.25062558	π-π bonded
trimer 167[a]	-922.81654848	-922.81728972	-922.82325975	0.25047492	π-π bonded
trimer 168[a]	-922.81580382	-922.81655142	-922.82258486	0.25020660	π-π bonded
trimer 169[a]	-922.81580574	-922.81653674	-922.82250632	0.25001098	π-π bonded
trimer 170[a]	-922.81701323	-922.81775741	-922.82354298	0.25100053	π-π bonded
trimer 171[a]	-922.81723784	-922.81800800	-922.82388164	0.25120527	π-π bonded
trimer 172[a]	-922.81613688	-922.81685744	-922.82267968	0.24992686	π-π bonded
trimer 173[a]	-922.81616468	-922.81689992	-922.82284763	0.25058144	π-π bonded
trimer 174[a]	-922.81622942	-922.81696732	-922.82287874	0.25019168	π-π bonded

	Tetramer				
Name	$E_{gas}^{\mathrm{N07Tdiff}}$(CP)	$E_{gas}^{\mathrm{N07Tdiff}}$	$E_{CCl_4}^{\mathrm{N07Tdiff}}$	$\delta G_{i,VRT}^{*,\mathrm{N07D}}$	Pattern
tetramer 1[a]	-1230.44483916	-1230.44627879	-1230.45050156	0.34427637	Dimer-like
tetramer 2[a]	-1230.44455905	-1230.44600100	-1230.45092382	0.34483743	Dimer-like
tetramer 3[a]	-1230.44455585	-1230.44599828	-1230.45027199	0.34466565	Dimer-like
tetramer 4[a]	-1230.44305368	-1230.44450151	-1230.44945402	0.34399868	Dimer-like
tetramer 5[a]	-1230.44327616	-1230.44467450	-1230.44936642	0.34522636	Dimer-like
tetramer 6[a]	-1230.43482381	-1230.43612487	-1230.44210178	0.34293216	Dimer-like
tetramer 7[a]	-1230.43048378	-1230.43176275	-1230.43907678	0.34275859	Dimer-like

Name	$E_{gas}^{\text{N07Tdiff}}$(CP)	$E_{gas}^{\text{N07Tdiff}}$	$E_{CCl_4}^{\text{N07Tdiff}}$	$\delta G_{i,VRT}^{*,\text{N07D}}$	Pattern
tetramer 8[a]	-1230.42811037	-1230.42916190	-1230.43703471	0.34057439	Dimer-like
tetramer 9[a]	-1230.43908809	-1230.44041721	-1230.44583712	0.34343690	Dimer-like
tetramer 10[a]	-1230.43881805	-1230.44013146	-1230.44561795	0.34372296	Dimer-like
tetramer 11[a]	-1230.43346565	-1230.43476336	-1230.44145220	0.34255443	Dimer-like
tetramer 12[a]	-1230.43742562	-1230.43871196	-1230.44437514	0.34330810	Dimer-like
tetramer 13[a]	-1230.43857901	-1230.43987744	-1230.44555241	0.34422895	Dimer-like
tetramer 14[a]	-1230.42093022	-1230.42176121	-1230.43062552	0.33747791	Dimer-like
tetramer 15[a]	-1230.43810748	-1230.43949039	-1230.44560838	0.34268102	Dimer-like
tetramer 16[a]	-1230.43257674	-1230.43379338	-1230.44071191	0.34142620	Dimer-like
tetramer 17[a]	-1230.43888754	-1230.44019250	-1230.44569228	0.34308800	Dimer-like
tetramer 18[a]	-1230.43159619	-1230.43263114	-1230.43827590	0.34102648	Dimer-like
tetramer 19[a]	-1230.43641457	-1230.43766404	-1230.44331827	0.34230979	Dimer-like
tetramer 20[a]	-1230.42992184	-1230.43106348	-1230.43799942	0.34099860	Dimer-like
tetramer 21[a]	-1230.42696127	-1230.42809345	-1230.43487487	0.34186646	Dimer-like
tetramer 22[a]	-1230.43727716	-1230.43853789	-1230.44414115	0.34295850	Dimer-like
tetramer 23[a]	-1230.42719704	-1230.42825513	-1230.43613787	0.34025082	Dimer-like
tetramer 24[a]	-1230.42757013	-1230.42861433	-1230.43614169	0.34073557	Dimer-like
tetramer 25[a]	-1230.42883484	-1230.42992343	-1230.43752308	0.34063376	Dimer-like
tetramer 26[a]	-1230.43103875	-1230.43218076	-1230.43907672	0.34052360	Dimer-like
tetramer 27[a]	-1230.43011359	-1230.43125358	-1230.43818973	0.34079897	Dimer-like
tetramer 28[a]	-1230.42465373	-1230.42568475	-1230.43405597	0.34093149	Dimer-like
tetramer 29[a]	-1230.43902313	-1230.44039373	-1230.44607836	0.34401008	Dimer-like
tetramer 30[a]	-1230.42620132	-1230.42728904	-1230.43535897	0.34130941	Dimer-like
tetramer 31[a]	-1230.43168503	-1230.43289802	-1230.44003856	0.34238386	Dimer-like
tetramer 32[a]	-1230.43452706	-1230.43591208	-1230.44226075	0.34428183	Dimer-like
tetramer 33[a]	-1230.43349639	-1230.43479366	-1230.44228183	0.34349834	Dimer-like
tetramer 34[a]	-1230.43387808	-1230.43515586	-1230.44140322	0.34270428	Dimer-like
tetramer 35[a]	-1230.43555614	-1230.43680255	-1230.44235029	0.34265326	Dimer-like
tetramer 36[a]	-1230.43303763	-1230.43433249	-1230.44164187	0.34397591	Dimer-like
tetramer 37[a]	-1230.43806541	-1230.43931281	-1230.44440758	0.34235358	Dimer-like
tetramer 38[a]	-1230.43424990	-1230.43549998	-1230.44120576	0.34291612	Dimer-like
tetramer 39[a]	-1230.43863093	-1230.44003195	-1230.44684465	0.34514546	Dimer-like
tetramer 40[a]	-1230.43334693	-1230.43465823	-1230.44229165	0.34345449	Dimer-like
tetramer 41	-1230.44719102	-1230.44853164	-1230.45310202	0.34360990	Double dimer
tetramer 42	-1230.44588338	-1230.44724643	-1230.45193549	0.34437453	Double dimer
tetramer 43	-1230.44646424	-1230.44789541	-1230.45296885	0.34548613	Double dimer
tetramer 44	-1230.44612017	-1230.44750223	-1230.45199353	0.34481550	Double dimer
tetramer 45	-1230.44456828	-1230.44582995	-1230.45073134	0.34374036	Double dimer
tetramer 46	-1230.44561750	-1230.44702261	-1230.45225039	0.34521379	Double dimer
tetramer 47	-1230.44497206	-1230.44627372	-1230.45094373	0.34399606	Double dimer
tetramer 48	-1230.44515993	-1230.44655420	-1230.45092553	0.34433941	Double dimer

Name	$E_{gas}^{\mathrm{N07Tdiff}}$(CP)	$E_{gas}^{\mathrm{N07Tdiff}}$	$E_{CCl_4}^{\mathrm{N07Tdiff}}$	$\delta G_{i,VRT}^{*,\mathrm{N07D}}$	Pattern
tetramer 49	-1230.44571272	-1230.44704079	-1230.45200809	0.34546909	Double dimer
tetramer 50	-1230.44373015	-1230.44513380	-1230.45130701	0.34482214	Double dimer
tetramer 51	-1230.44346207	-1230.44489524	-1230.45058497	0.34459429	Double dimer
tetramer 52	-1230.44357147	-1230.44496157	-1230.45121013	0.34527770	Double dimer
tetramer 53	-1230.44452617	-1230.44590024	-1230.45019998	0.34433186	Double dimer
tetramer 54	-1230.44408765	-1230.44547339	-1230.45012027	0.34458995	Double dimer
tetramer 55	-1230.44510841	-1230.44651230	-1230.45068776	0.34527460	Double dimer
tetramer 56	-1230.44448754	-1230.44588102	-1230.45016145	0.34495411	Double dimer
tetramer 57	-1230.44421489	-1230.44557570	-1230.45015472	0.34486504	Double dimer
tetramer 58	-1230.44416299	-1230.44553265	-1230.45012500	0.34503281	Double dimer
tetramer 59[a]	-1230.43770316	-1230.43895768	-1230.44489459	0.34287653	Double dimer
tetramer 60[a]	-1230.43252057	-1230.43355288	-1230.44027635	0.34116822	Double dimer
tetramer 61[a]	-1230.44921715	-1230.45062883	-1230.45465191	0.34465679	Trimer-like linear
tetramer 62[a]	-1230.44847206	-1230.44988354	-1230.45406107	0.34524709	Trimer-like linear
tetramer 63	-1230.44526255	-1230.44660940	-1230.45113045	0.34352038	Trimer-like linear
tetramer 64	-1230.44552173	-1230.44685169	-1230.45153066	0.34421082	Trimer-like linear
tetramer 65	-1230.44389732	-1230.44523266	-1230.45014745	0.34314161	Trimer-like linear
tetramer 66	-1230.44638646	-1230.44779821	-1230.45205653	0.34537755	Trimer-like linear
tetramer 67[a]	-1230.44574196	-1230.44710640	-1230.45161028	0.34496770	Trimer-like linear
tetramer 68[a]	-1230.44458606	-1230.44601799	-1230.45061737	0.34455872	Trimer-like linear
tetramer 69	-1230.44361378	-1230.44493293	-1230.45016223	0.34432671	Trimer-like linear
tetramer 70	-1230.44563747	-1230.44703026	-1230.45136335	0.34583781	Trimer-like linear
tetramer 71	-1230.44701168	-1230.44842247	-1230.45258795	0.34691152	Trimer-like linear
tetramer 72[a]	-1230.44405062	-1230.44540954	-1230.44987574	0.34497216	Trimer-like linear
tetramer 73[a]	-1230.44093930	-1230.44217744	-1230.44759155	0.34348956	Trimer-like linear
tetramer 74[a]	-1230.44141857	-1230.44278139	-1230.44861835	0.34470556	Trimer-like linear
tetramer 75[a]	-1230.44150715	-1230.44277970	-1230.44842271	0.34427394	Trimer-like linear
tetramer 76[a]	-1230.44104885	-1230.44234850	-1230.44798311	0.34411915	Trimer-like linear
tetramer 77[a]	-1230.44128760	-1230.44262072	-1230.44848810	0.34463196	Trimer-like linear
tetramer 78[a]	-1230.43469458	-1230.43581986	-1230.44132633	0.34213842	Trimer-like linear
tetramer 79[a]	-1230.43973750	-1230.44114336	-1230.44794092	0.34444778	Trimer-like linear
tetramer 80[a]	-1230.43856331	-1230.43973704	-1230.44498064	0.34305799	Trimer-like linear
tetramer 81[a]	-1230.42950801	-1230.43062915	-1230.43791970	0.34323864	Trimer-like linear
tetramer 82[a]	-1230.44033466	-1230.44164849	-1230.44700952	0.34431668	Trimer-like linear
tetramer 83[a]	-1230.44202189	-1230.44337580	-1230.44866678	0.34491558	Trimer-like linear
tetramer 84[a]	-1230.44145854	-1230.44277080	-1230.44758179	0.34417405	Trimer-like linear
tetramer 85	-1230.44449286	-1230.44578562	-1230.45024376	0.34428781	Trimer-like cycle
tetramer 86	-1230.44484580	-1230.44621860	-1230.45116909	0.34573397	Trimer-like cycle
tetramer 87	-1230.44474540	-1230.44611109	-1230.45024995	0.34496939	Trimer-like cycle
tetramer 88[a]	-1230.44337707	-1230.44469529	-1230.44911242	0.34456382	Trimer-like cycle
tetramer 89[a]	-1230.43847902	-1230.43974826	-1230.44518821	0.34399292	Trimer-like cycle

Name	$E_{gas}^{\mathrm{N07Tdiff}}$(CP)	$E_{gas}^{\mathrm{N07Tdiff}}$	$E_{CCl_4}^{\mathrm{N07Tdiff}}$	$\delta G_{i,VRT}^{*,\mathrm{N07D}}$	Pattern
tetramer 90	-1230.44376598	-1230.44511139	-1230.45024936	0.34458731	Star
tetramer 91	-1230.44997937	-1230.45135668	-1230.45560817	0.34577324	Linear
tetramer 92	-1230.44876981	-1230.45002233	-1230.45429171	0.34450402	Linear
tetramer 93	-1230.44952655	-1230.45092399	-1230.45491862	0.34504753	Linear
tetramer 94	-1230.45015610	-1230.45159623	-1230.45548897	0.34594815	Linear
tetramer 95	-1230.44820214	-1230.44943078	-1230.45369003	0.34450887	Linear
tetramer 96	-1230.44938371	-1230.45078203	-1230.45489639	0.34530619	Linear
tetramer 97	-1230.44769539	-1230.44889957	-1230.45308392	0.34355703	Linear
tetramer 98	-1230.44900244	-1230.45035072	-1230.45470565	0.34567101	Linear
tetramer 99	-1230.44912876	-1230.45045893	-1230.45455491	0.34532617	Linear
tetramer 100	-1230.44901948	-1230.45038376	-1230.45449559	0.34527443	Linear
tetramer 101	-1230.44802125	-1230.44932155	-1230.45371479	0.34471340	Linear
tetramer 102	-1230.44756636	-1230.44891104	-1230.45331467	0.34463615	Linear
tetramer 103	-1230.44763764	-1230.44885781	-1230.45294164	0.34408702	Linear
tetramer 104	-1230.44965353	-1230.45102374	-1230.45501076	0.34605933	Linear
tetramer 105	-1230.44650561	-1230.44786665	-1230.45225842	0.34528195	Linear
tetramer 106	-1230.44955423	-1230.45093562	-1230.45507142	0.34655487	Linear
tetramer 107	-1230.44711040	-1230.44836902	-1230.45267643	0.34473481	Linear
tetramer 108	-1230.44753750	-1230.44895250	-1230.45374082	0.34609478	Linear
tetramer 109	-1230.44874034	-1230.45000858	-1230.45413844	0.34406437	Linear
tetramer 110	-1230.44259154	-1230.44384988	-1230.44933445	0.34569135	Linear
tetramer 111	-1230.44631130	-1230.44768130	-1230.45206770	0.34528213	Linear
tetramer 112	-1230.44316393	-1230.44443392	-1230.45075981	0.34413364	Linear
tetramer 113	-1230.44599494	-1230.44744377	-1230.45165054	0.34548724	Linear
tetramer 114	-1230.44582728	-1230.44715973	-1230.45181653	0.34591448	Linear
tetramer 115	-1230.44207739	-1230.44332464	-1230.44920556	0.34440638	Linear
tetramer 116	-1230.44383400	-1230.44512924	-1230.45111756	0.34551346	Linear
tetramer 117	-1230.44480100	-1230.44616461	-1230.45056428	0.34485984	Linear
tetramer 118	-1230.44360310	-1230.44497175	-1230.45052211	0.34558650	Linear
tetramer 119	-1230.44427396	-1230.44567936	-1230.45066142	0.34570494	Linear
tetramer 120	-1230.44265621	-1230.44408095	-1230.45075037	0.34568025	Linear
tetramer 121	-1230.44764052	-1230.44912874	-1230.45444437	0.34718575	Linear
tetramer 122	-1230.44323022	-1230.44463901	-1230.45116614	0.34653534	Linear
tetramer 123	-1230.44380869	-1230.44512916	-1230.44966441	0.34506904	Linear
tetramer 124	-1230.44234614	-1230.44369036	-1230.44961707	0.34533319	Linear
tetramer 125	-1230.44534041	-1230.44662772	-1230.45181527	0.34557242	Linear
tetramer 126	-1230.45088562	-1230.45232332	-1230.45609363	0.34600237	Linear
tetramer 127	-1230.44364223	-1230.44494743	-1230.44959549	0.34524913	Cocktail
tetramer 128	-1230.45003109	-1230.45116314	-1230.45510654	0.34432825	Cyclic
tetramer 129	-1230.45007154	-1230.45122089	-1230.45513005	0.34452997	Cyclic
tetramer 130	-1230.45110144	-1230.45236563	-1230.45621025	0.34549853	Cyclic

Name	$E_{gas}^{\text{N07Tdiff}}$(CP)	$E_{gas}^{\text{N07Tdiff}}$	$E_{CCl_4}^{\text{N07Tdiff}}$	$\delta G_{i,VRT}^{*,\text{N07D}}$	Pattern
tetramer 131	-1230.45103259	-1230.45228199	-1230.45606134	0.34537629	Cyclic
tetramer 132	-1230.45162876	-1230.45288154	-1230.45670672	0.34629545	Cyclic
tetramer 133	-1230.45174083	-1230.45306364	-1230.45689608	0.34652579	Cyclic
tetramer 134	-1230.45148295	-1230.45277516	-1230.45666049	0.34632990	Cyclic
tetramer 135	-1230.45179670	-1230.45310712	-1230.45704117	0.34703327	Cyclic
tetramer 136	-1230.45012189	-1230.45144529	-1230.45521507	0.34732955	Cyclic
tetramer 137[a]	-1230.44280637	-1230.44422278	-1230.44888824	0.34416165	π-π bonded
tetramer 138[a]	-1230.43408329	-1230.43541601	-1230.44124770	0.34217702	π-π bonded
tetramer 139[a]	-1230.41654195	-1230.41749365	-1230.42638449	0.33874190	π-π bonded
tetramer 140[a]	-1230.43044733	-1230.43161799	-1230.43761045	0.34059036	π-π bonded
tetramer 141[a]	-1230.43081754	-1230.43207971	-1230.43891683	0.34105269	π-π bonded
tetramer 142[a]	-1230.43497701	-1230.43622250	-1230.44219003	0.34098248	π-π bonded
tetramer 143[a]	-1230.42820381	-1230.42939796	-1230.43653581	0.34099729	π-π bonded
tetramer 144[a]	-1230.43157519	-1230.43285607	-1230.43970529	0.34157030	π-π bonded

[a] Obtained from the sampling over MD trajectories.

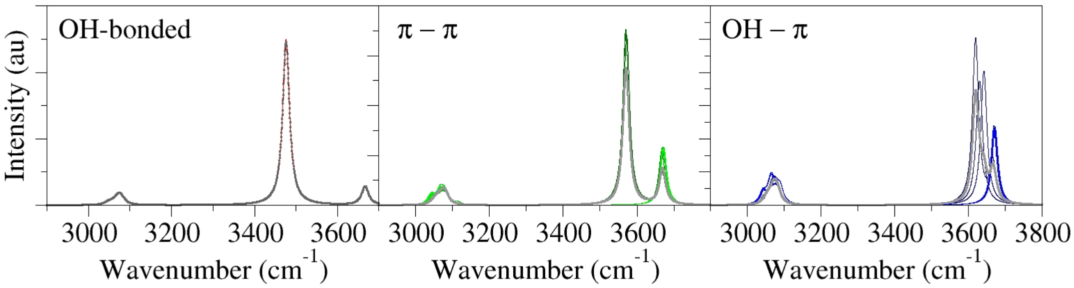

Figure S12: IR spectra of individual dimer conformers within each interaction pattern, with the average spectrum for each pattern depicted in gray.

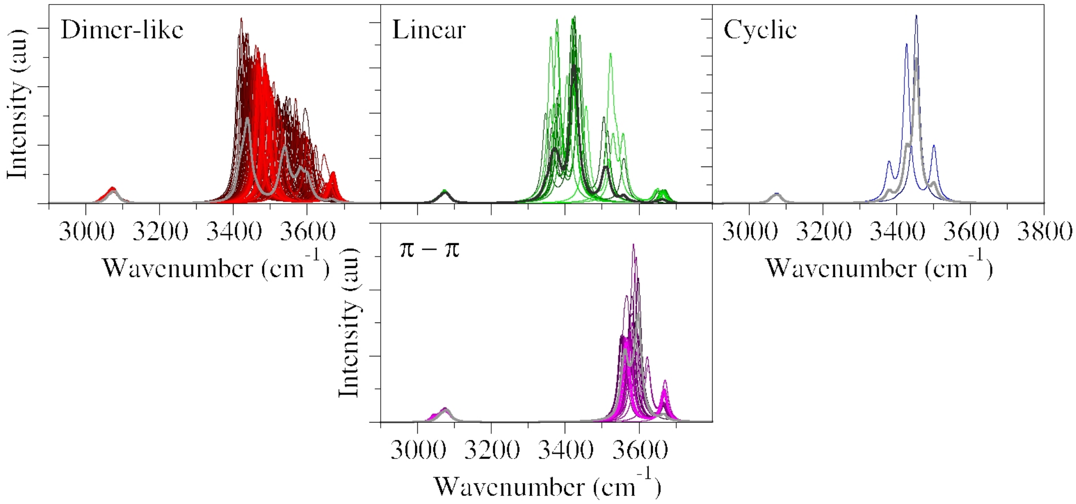

Figure S13: IR spectra of individual trimer conformers within each interaction pattern, with the average spectrum for each pattern depicted in gray.

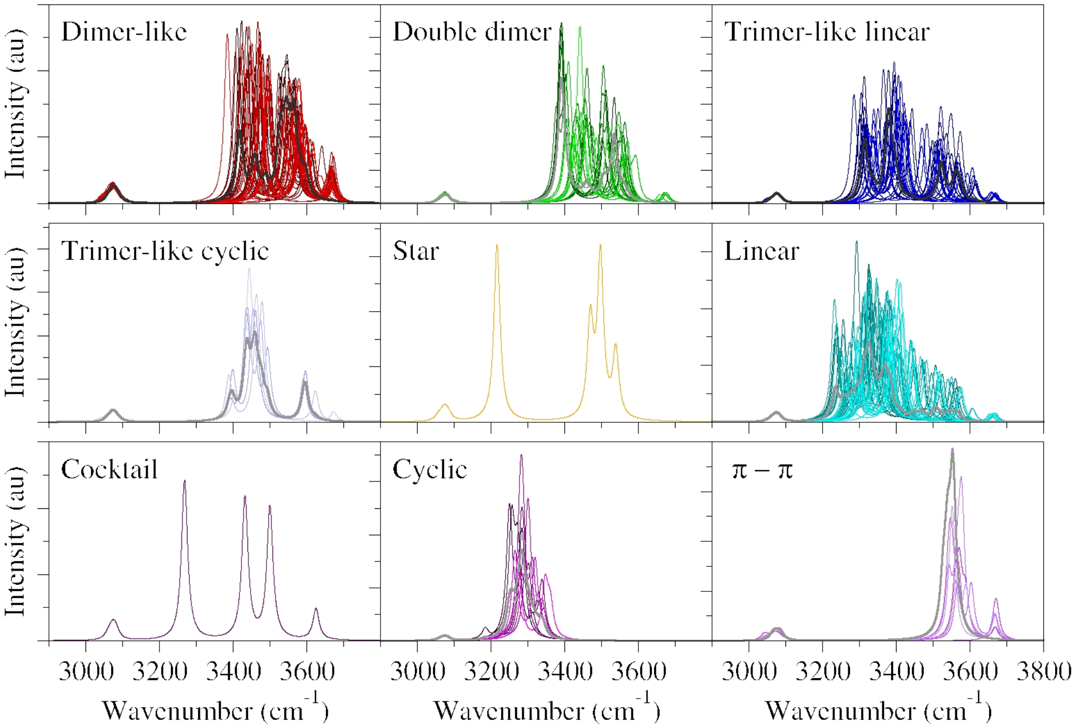

Figure S14: IR spectra of individual tetramer conformers within each interaction pattern, with the average spectrum for each pattern depicted in gray.

G Interaction patterns

Figures S15 and S16 present the results for the distinct interaction patterns identified in trimers and tetramers, respectively.

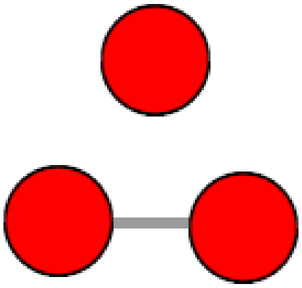

(a) Dimer-like

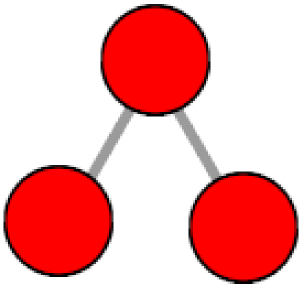

(b) Linear

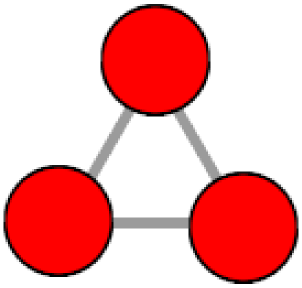

(c) Cyclic

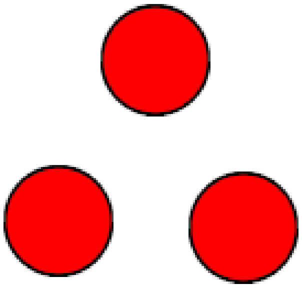

(d) $\pi - \pi$ bonded

Figure S15: Interaction patterns for trimers.

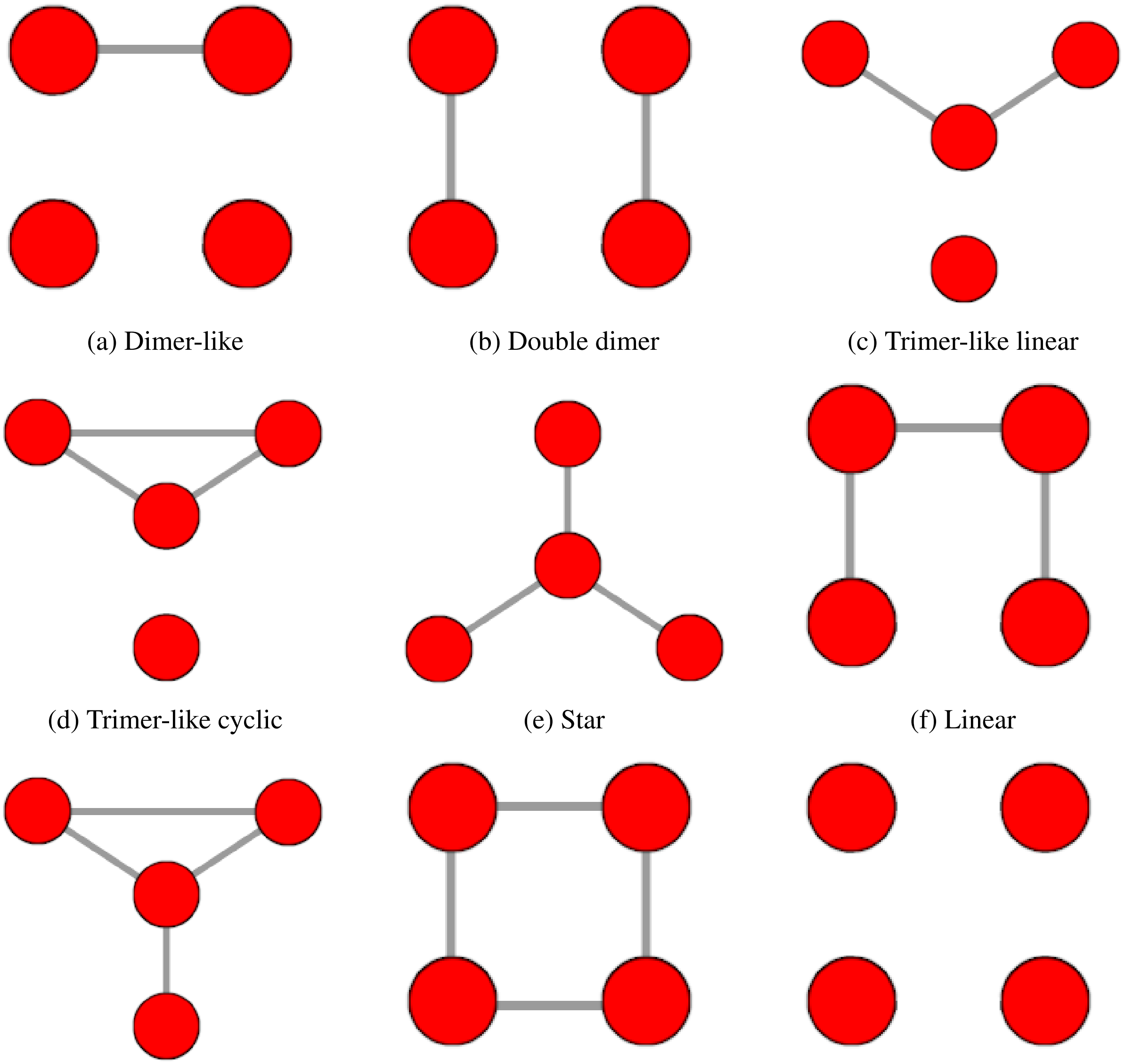

(a) Dimer-like (b) Double dimer (c) Trimer-like linear

(d) Trimer-like cyclic (e) Star (f) Linear

(g) Cocktail (h) Cyclic (i) π-π bonded

Figure S16: Interaction patterns for trimers.

H Equilibrium properties

To provide a comprehensive analysis of the equilibrium properties, Table S6 presents the equilibrium constants derived using the **C/d** and **Q/s** methodologies, compared to the wide range of values reported in the literature. These values were obtained using different threshold sets and conformer sets, respectively, highlighting the influence of methodological parameters on

the calculated constants. Values of the **C/d** and **Q/s** equilibrium constants employed throughout this work, computed relative to a single monomer unit, are presented in Table S7 to facilitate comparison of the relative stability of the aggregates. Additionally, Table S8 compares the equilibrium concentrations measured during the MD simulations–obtained from snapshots analyzed at two different intervals (1.0 ps and 5.0 ps)–with those calculated using the **C/d** method to asses the validity of the monomer-dimer-trimer-tetramer equilibrium model.

Table S6: Equilibrium constants derived from the **C/d** and **Q/s** methodologies obtained with different thresholds and conformer sets, respectively.

	**C/d**	Loose	**Q/s**	Small	Experiment
Dimer	0.55271	0.67757	0.02366	0.02366	1.39[a],1.26[b],0.7[e],0.93[e], 0.7[f],0.74[h],0.94[i],1.5[m], 1.35[o],3.2[o]
Trimer	0.68544	1.03183	0.01136	0.01102	4.1[a],4.78[c],13.6[d],8.9[d], 4.1[g],3.1[i],14.9[j],5.6[j], 12.7[j],11.27[k],2.97[l], 2.1[n],2.4[n]
Tetramer	1.22125	2.16618	0.06867	0.06551	12.01[a],10.1[i],3.3[n],3.4[n]

[a] Reference [5]. [b] Reference [6]. [c] Reference [7]. [d] Reference [8]. [e] Reference [9]. [f] Reference [10]. [g] Reference [11]. [h] Reference [12]. [i] Reference [13]. [j] Reference [14]. [k] Reference [15]. [l] Reference [16]. [m] Reference [17]. [n] Reference [18]. [o] Reference [19].

Table S7: Equilibrium constants derived from the **C/d** and **Q/s** methodologies refered to the same stoichiometric coefficient for the momoner, which is set to one. I.e. we refer the constants to the following chemical equation: $(PhOH)_1 \rightarrow \frac{1}{n}(PhOH)_n$. These constants and thus related to those in Table S6 as the n-th roots of the latter.

	Dimer	Trimer	Tetramer
**C/d**	0.74344	0.88171	1.05124
**Q/s**	0.15382	0.22480	0.51191

Table S8: Comparison of equilibrium concentrations from MD simulations (using two different snapshot frequencies) and the **C/d** method.

	MD (1.0 ps)			
C_0	Monomer	Dimer	Trimer	Tetramer
0.11224102	0.0988367	0.0053250	0.0006830	0.0001389
0.42596327	0.2615270	0.0380429	0.0126644	0.0061371
0.98592409	0.3809290	0.0807963	0.0366411	0.0238063
	MD (5.0 ps)			
C_0	Monomer	Dimer	Trimer	Tetramer
0.11224013	0.0988713	0.0053066	0.0006822	0.0001397
0.42595317	0.2614950	0.0380395	0.0126967	0.0061513
0.98593149	0.3808990	0.0807995	0.0366851	0.0237729
	**C/d**			
C_0	Monomer	Dimer	Trimer	Tetramer
0.11224102	0.0989556	0.0054123	0.0006642	0.0001171
0.42596327	0.2736511	0.0413897	0.0140463	0.0068485
0.98592409	0.4344878	0.1043404	0.0562217	0.0435226

References

[1] Brehm, M.; Thomas, M.; Gehrke, S.; Kirchner, B. TRAVIS—A free analyzer for trajectories from molecular simulation *J. Chem. Phys.* **2020**, *152*, 164105.

[2] Scott, A. P.; Radom, L. Harmonic vibrational frequencies: An evaluation of Hartree-Fock, Möller-Plesset, quadratic configuration interaction, density functional theory, and semiempirical scale factors *J. Phys. Chem.* **1996**, *100*, 16502–16513.

[3] Roth, W.; Imhof, P.; Gerhards, M.; Schumm, S.; Kleinermanns, K. Reassignment of ground and first excited state vibrations in phenol *Chem. Phys.* **2000**, *252*, 247.

[4] Lampert, H.; Mikenda, W.; Karpfen, A. Molecular geometries and vibrational spectra of phenol, benzaldehyde, and salicylaldehyde: Experimental versus quantum chemical data *J. Phys. Chem.* **1997**, *101*, 2254.

[5] Coggeshall, N. D.; Saier, E. L. Infrared Absorption Study of Hydrogen Bonding Equilibria *J. Am. Chem. Soc.* **1951**, *73*, 5414–5418.

[6] Huggins, C. M.; Pimentel, G. C.; Shoolery, J. N. Proton Magnetic Resonance Studies of the Hydrogen Bonding of Phenol, Substituted Phenols and Acetic Acid. *J. Phys. Chem.* **1956**, *60*, 1311–1315.

[7] Saunders, M.; Hyne, J. B. Study of hydrogen bonding in systems of hydroxylic compounds in carbon tetrachloride through the use of NMR *J. Chem. Phys.* **1958**, *29*, 1319.

[8] Pimentel, G. C. Infrared spectroscopy: A chemist's tool *J. Chem. Educ.* **1960**, *37*, 651.

[9] Maguire, M. M.; West, R. Hydrogen-bonding studies VII. Near infrared spectroscopic studies of the intermolecular hydrogen bonding of phenol, p-cresol and p-chlorophenol *Spectrochim. Acta* **1961**, *17*, 369.

[10] Powell, D. L.; West, R. Hydrogen bonding studies VIII. The thermodynamics of hydrogen bonding of phenol to some oxygen-containing molecules and to benzene *Spectrochim. Acta* **1964**, *20*, 983 – 991.

[11] Johnson, J. R.; Christian, S. D.; Affsprung, H. E. 1. Self-association and hydration of phenol in carbon tetrachloride *J. Chem. Soc.* **1965**, 1–6.

[12] Singh, S.; Rao, C. N. R. Deuterium isotope effects on hydrogen bonding *Can. J. Chem.* **1966**, *44*, 2611–2615.

[13] Whetsel, K. B.; Lady, J. H. Spectrometry of fuels. Chapter 20: Self-association of phenol in nonpolar solvents; Springer, 1970.

[14] Woolley, E.; Travers, J. G.; Erno, B. P.; Hepler, L. G. Molecular association of hydrogen-bonding solutes. Phenol in carbon tetrachloride *J. Phys. Chem.* **1971**, *75*, 3591.

[15] Kimtys, L.; Mikulskis, P.; Shapet'ko, N. N. A NMR study of phenol self-association. The quasi-chemical approximation *Magn. Reson. Chem.* **1973**, *5*, 361.

[16] Schaefer, T.; Rowbotham, J. B.; Chum, K. The proton magnetic resonance spectra of phenol in the absence of intermolecular proton exchange *Can. J. Chem.* **1976**, *54*, 3666–3671.

[17] Seidel, H.; Ritter, C.; Fruwert, J.; Geiseler, G. Über die substituentenabhängigkeit der Donatorund Akzeptoreigenschaften der Hydroxylgruppe in parasubstituierten Phenolen und Phenol—Anisol-Komplexen *Spectrochim. Acta Part A* **1976**, *32*, 705–708.

[18] Lin, L.-N.; Christian, S. D.; Tucker, E. E. Solute activity study of the self-association of phenol in cyclohexane and carbon tetrachloride *J. Phys. Chem.* **1978**, *82*, 1897.

[19] Josefiak, C.; Schneider, G. Determination of reaction volumes of hydrogen-bonding equilibria by high-pressure near-infrared spectroscopy. 2. Self-association of phenol in CCl4 up to 1 kbar *J. Phys. Chem.* **1980**, *84*, 3004.
